# Indole-based FLT3 inhibitors and related scaffolds as potential therapeutic agents for acute myeloid leukemia

**DOI:** 10.1186/s13065-023-00981-8

**Published:** 2023-07-12

**Authors:** Hend A. A. Ezelarab, Taha F. S. Ali, Samar H. Abbas, Heba A. Hassan, Eman A. M. Beshr

**Affiliations:** Department of Medicinal Chemistry, Faculty of Pharmacy, Minia University, Minia, 61519 Egypt

**Keywords:** Acute myeloid leukemia, FLT3 inhibitors, Indoles, Indole analogues

## Abstract

Fms-like tyrosine kinase 3 (FLT3) mutation mechanisms are among the most common genetic abnormalities detected in about 30% of acute myeloid leukemia (AML) patients. These mutations are accompanied by poor clinical response, although all these progressions in identifying and interpreting biological AML bio-targets. Several small structured FLT3 inhibitors have been ameliorated to struggle against AML. Despite all these developments regarding these inhibitors, the Overall survival rate is about five years or more in less than one-third of diagnosed AML patients. Midostaurin was the first FDA-approved FLT3 inhibitor in 2017 in the United States and Europe for AML remedy. Next, Gilteritinib was an FDA-approved FLT3 inhibitor in 2018 and in the next year, Quizartinib was approved an as FLT3 inhibitor in Japan. Interestingly, indole-based motifs had risen as advantaged scaffolds with unusual multiple kinase inhibitory activity. This review summarises indole-based FLT3 inhibitors and related scaffolds, including FDA-approved drugs, clinical candidates, and other bioactive compounds. Furthermore, their chemotypes, mechanism of action, and interaction mode over both wild and mutated FLT3 target proteins had been judgmentally discussed. Therefore, this review could offer inspiring future perspectives into the finding of new FLT3-related AML therapies.

## Introduction

Acute myeloid leukemia (AML) stands among the utmost widely distributed kinds of hematological cancers, which represents an aggressive clonal syndrome in hematological stem cells (HSCs) in several tissues such as bone marrow, peripheral blood vessels, etc. [1]. Unwell-discriminated proliferating cells can restrict normal hematological proliferation operation, resulting in dangerous infections, anemia, and serious bleeding [2]. Relying on previous literature studies, it was found that the survival of patients identified with AML within five years of infection was fewer than 50%. The mortality rate of old-aged patients was higher than half of the detected AML infections in the west [3, 4]. By 2020, AML became an essential health concern as it is responsible for the highest mortality rate in the USA, reach to eleven thousand yearly [5]. Long-term therapy of AML with Cytarabine and Anthracycline was unsatisfactory as the remedy rate of the older patients didn't exceed 5–15%, triggering the development of effective candidates targeting AML genetic mediators [6]. Hot genomic studies discovered that Fms-like tyrosine kinase 3 (FLT3) was overexpressed in AML leukemic blasts, and the FLT3 gene mutation was highly common in nearly 30% of AML-investigated patients. The percentages of the most common mutations FLT3-ITD and point mutations within the tyrosine kinase domain (TKD) were 20–25% and 5–10%, respectively [7–9]. Herein, FLT3 has become an outstanding therapeutic target for AML therapy, guiding the development of various small molecule inhibitors of FLT3, such as Lestaurtinib, Midostaurin, and Quizartinib.

**Fig. 1:**
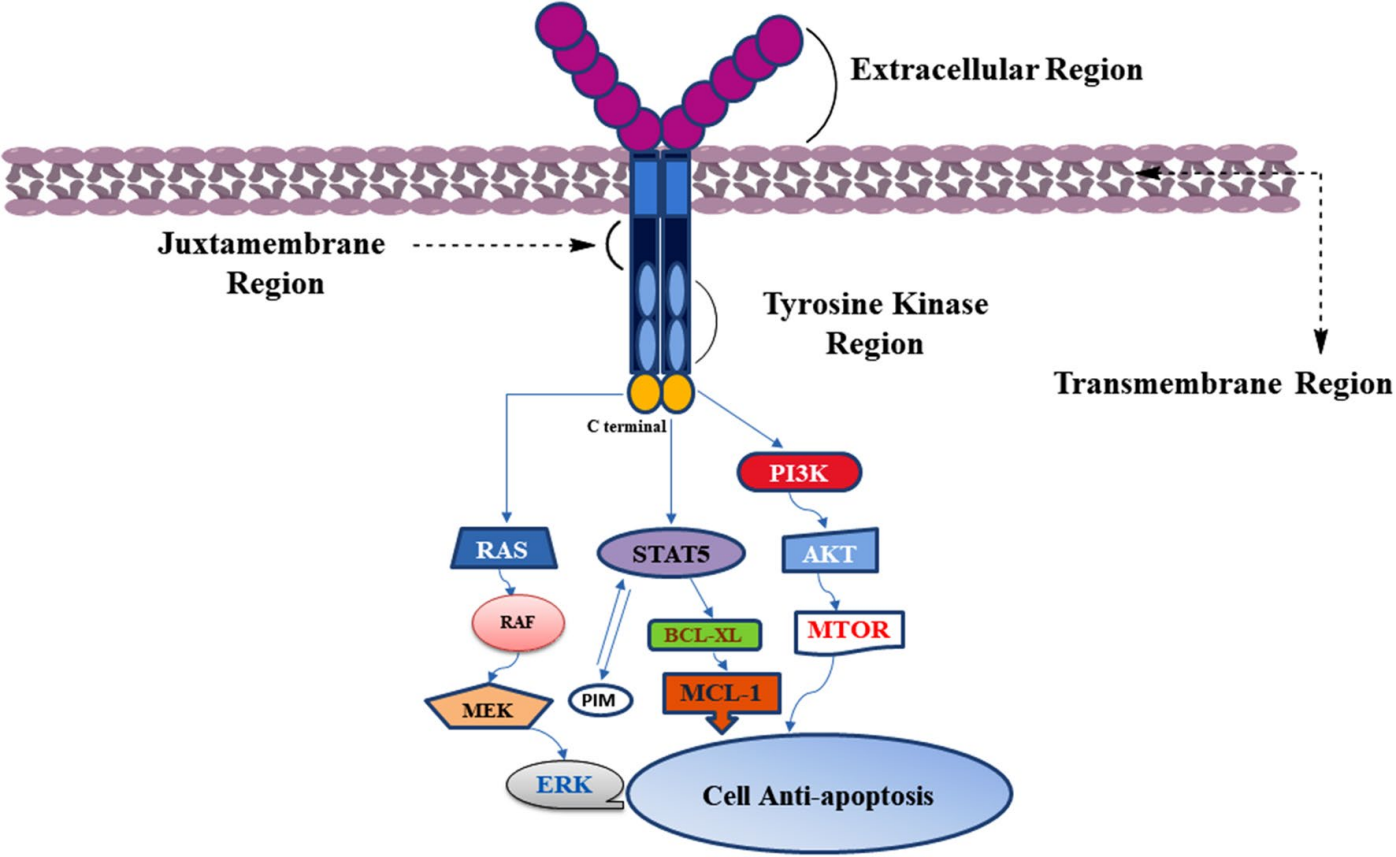
FLT3 receptor and its physiological downstream signaling cascades

FLT3, also known as CD135, is a cytokine receptor belonging to the receptor tyrosine kinase (RTK) class III family and exhibits vital accountability for the HSCs' anti-apoptotic propagation and discrimination [10]. FLT3 is extremely like c-Kit, c- Fms, and PDGFR, likewise important members of the RTK family. FLT3 consists of five loops of immunoglobulin in the extracellular region, a short *α*-helix transmembrane region, a juxtamembrane region, two regions of tyrosine kinase separated by inserted zones, and an intracellular C-terminal region (Fig. 1) [11, 12]. The intracellular region is encompassed by a shortened irregularly structured linker (including residues 564–571), a juxtamembrane region (including residues 572–603), and a tyrosine kinase region [13]. Like the common physiological status, FLT3 is triggered by the conjugation of FLT3-ligand (FL), such as a hematopoietic growth factor, to immunoglobulins’ region D2 and D3, where a pair of the ligands interacts with the receptor leading to its dimerization. This FLT3 dimer transfers the signal to the intracellular region, where the tyrosine kinase regions of FLT3 are connected and phosphorylate themselves. FL excites the innate hematopoietic cells’ propagation by stimulating FLT3, which triggers many downstream colonial stimulating factors and interleukin mediators [14]. In the case of FL absence, the FLT3’s juxtamembrane region hampers the foundation and stimulation of FLT3 dimers under normal situations. Yet, the interaction of FL with FLT3 consequences in the formation of the receptor dimers and, at the same moment, tyrosine residues' autophosphorylation in the intracellular region, which triggers a group of signaling cascades through phosphorylation of subsequent down streaming proteins, including RAS/MAPK, JAK/STAT, and PI3K/AKT/mTOR paths [15–17].

Mutations for FLT3 inhibitors in AML patients may happen by on-target or off-target resistance mechanisms [18]. On-target mutation mechanisms against FLT3 inhibitors are represented by point mutations in the TKD of FLT3-ITD, such as the mutation within the gatekeeper F691L, which resulted in resistance versus all these inhibitors that are currently in clinical use and the activating kinase domain (KD) mutations D835V/Y/F and Y842C (also Y842H) that resulted in resistance against specifically type 2 FLT3 hampering agents [19–21]. The off-target mutation mechanisms are represented by the stimulation of FLT3 downstream signaling pathways, such as the JAK/STAT5 or PI3K/AKT/mTOR pathway in mutated FLT3 AML cells, and like these cascades resulted in lessening the cytotoxic efficacy of FLT3 inhibitors (such as Midostaurin, Sorafenib, and Quizartinib) on AML cell lines [22, 23]. Also, off-target mutations include specific mutations such as TET2, RAS, and IDH1/2, activation of SYK and AXL (like Midostaurin and Quizartinib), upregulation of Pim and FLT3 ligand (FL) (such as Lestaurtinib, Midostaurin, Sorafenib, and Quizartinib), and preservation of FLT3-ITD AML cells in bone marrow microenvironment (like Quizartinib, Sorafenib, and Gilteritinib) [24–28]. About 30% of recently identified AML cases were recently accompanied by FLT3 mutations [10]. Therefore, phase I/II clinical studies of the monotherapy protocols with the common FLT3 inhibitors (like Lestaurtinib, Midostaurin, and Quizartinib) in AML patients had shown a poor clinical response as the drug resistance rapidly developed after the starting of these treatment regimens owing to like these types of mutation [6]. Mutations in both FLT3-ITD and tyrosine kinase regions could facilitate the activation of FLT3 kinase in the absence of FL and consequently promote cell propagation and survival, resulting in AML. The phenomenon of point mutations’ incidence in the FLT3 juxtamembrane region (FLT3-JM-PMs) is another mode of FLT3 mutations, which affords lower levels of the receptor autophosphorylation and its down proceeding mediator STAT5 in comparison with FLT3-ITD and FLT3 tyrosine region mutations [28].

FLT3 was overexpressed in AML leukemic blasts, and the FLT3 gene mutation was highly common in nearly 30% of AML-investigated patients [10]. So, the inhibition of FLT3 and its downstream signaling mediators, including PI3K/Akt, MAPK/Erks, and STAT5, become highly promising strategies that could potentiate the abnormal growth of the leukemic blasts resulting in apoptosis induction [29]. Because of considering FLT3 as a promising therapeutic target for AML therapy, this leads to the discovery of various small-molecule FLT3 inhibitors, such as Midostaurin, Lestaurtinib, and Quizartinib [30]. However, drug mutation mechanisms appeared shortly after commencing AML therapy. Despite FLT3 circumvention, STAT5 and MAPK downstream pathways are kept active in some mutated FLT3 AML cases, proposing counteractive mechanisms such as FLT3-independent triggering STAT5 and MAPK pathways [31]. Other mutation mechanisms were suggested involving non-classical TKD mutation that resisted FLT3 inhibitors by stimulating other anti-apoptotic proteins such as the anti-apoptotic protein Mcl-1 [32]. The outcomes from clinical trials illustrate that FLT3 circumvention isn’t a useful strategy if used individually and that combinatorial therapeutic protocols of FLT3 inhibitors with other bioactive compounds affecting the apoptotic mechanisms could counteract drug mutation mechanisms and be clinically valuable for FLT3 harboring AML patients [18].

## Small structured FLT3 inhibitors

Because of dysregulation and highly frequent mutation mechanisms of FLT3 kinase in AML, this oncogenic agent has been considered an efficient drug target in AML [33, 34]. Small structured FLT3 inhibitors fit into the kinase domain of the FLT3 receptor to abolish autophosphorylation and downstream signaling cascades [35, 36]. Based on the site of interaction of the FLT3 inhibitors to the kinase regions, they were classified into type-I and type-II. Type I FLT3 inhibitors (including Midostaurin, Lestaurtinib, Gilteritinib, Crenolanib, and Sunitinib), which are ATP competitive agents that can distinguish the active conformation DFG-in homology of FLT3 (Asp-Phe-Gly, three residues related to the conformational alteration) targeting the ATP binding area, while type II FLT3 circumventing agents, (including Tandutinib, Sorafenib, and Quizartinib), are capable of binding into the inactive conformation DFG-out homology model of FLT3 and prevent the kinase stimulation through an additional hydrophobic region beside the ATP binding site [37, 38]. Compared to type I inhibitors, type II inhibitors commonly preserve strong selectivity due to the lower conserved hydrophobic region rather than the ATP region [39]. Briefly, type I FLT3 hampering agents are less specific towards FLT3 interacting with the DFG-in conformation (active conformation) and consequence in lower antileukemia efficacy through several RTKs’ circumvention [40–47]. Inversely, type II FLT3 hampering agents (Linifanib) have extra selectivity and potency interacting with the DFG-out conformation (inactive conformation), besides being characterized with lower off-target toxicity [35]. D835 is the most frequent FLT3-TKD mutation, and this resistance mechanism stabilizes FLT3 in the active conformation. This stabilization condition lessens the inhibitory activity of type-II FLT3 inhibitors, as the TK domain is more energetically stable, favoring being kept in the DFG-in conformation [21]. These mutation mechanisms do not affect the fitting of type-I inhibitors into the FLT3 kinase domain since these inhibitors fit into the active conformation of the FLT3 kinase [21]. Besides, small structured FLT3 inhibitors can be further categorized into reversible and irreversible inhibitors [13, 18]. This classification is based on making a covalent bond with C695 on the FLT3 kinase domain. For instance, FF-10101 is a new FLT3 inhibitor that covalently binds to the C695 cysteine moiety of the FLT3 kinase domain and can alleviate the on-target mutation mechanisms [48]. Preclinical research studies have evaluated that this inhibitor is kept efficient FLT3 inhibitor despite the existence of the F691L mutation, which highlights that irreversible inhibitors could be efficient therapeutic agents in drug-mutated mechanisms [48].

### Binding modes of FLT3 inhibitors

**Fig. 2:**
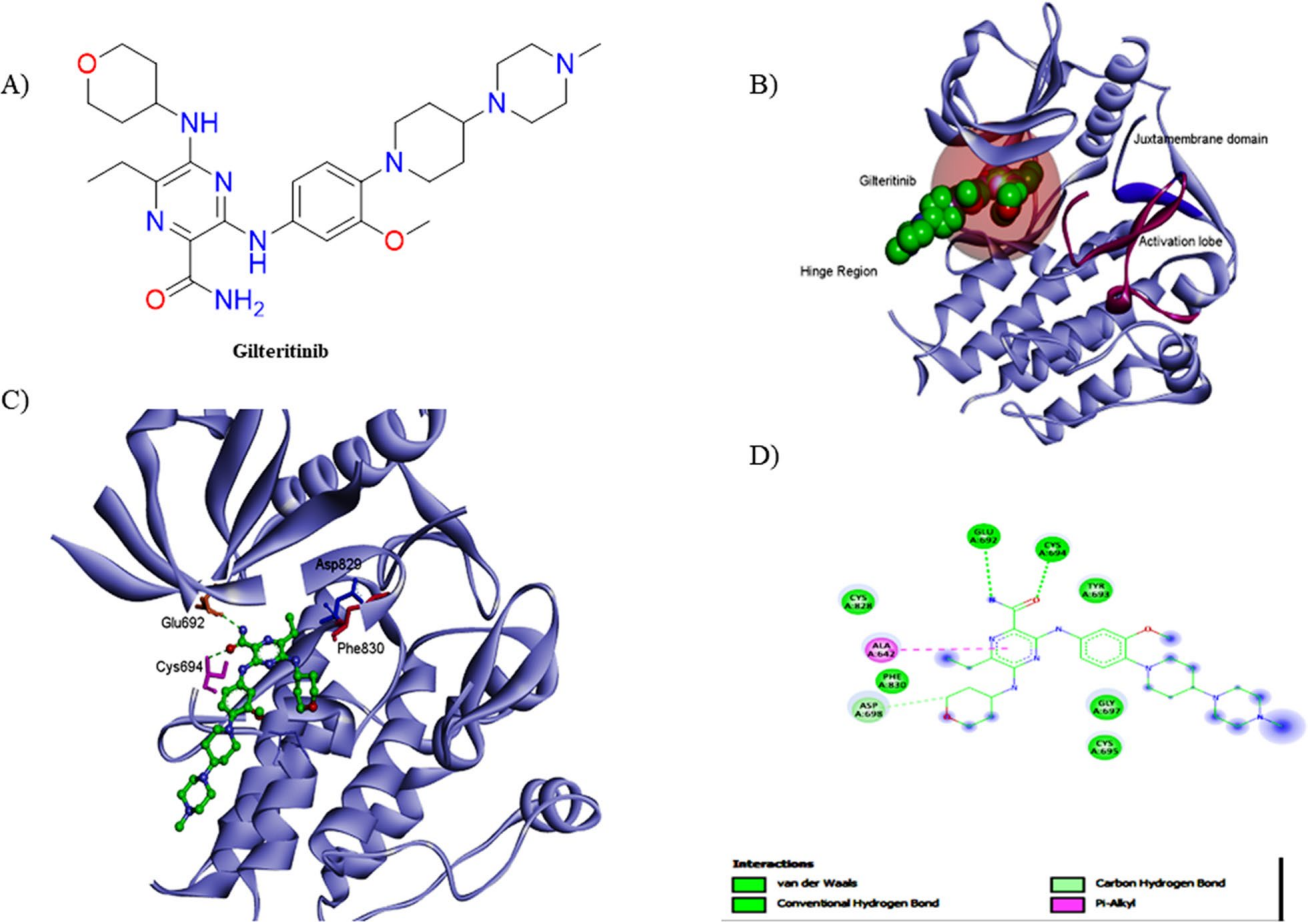
**A** The chemical structure of Gilteritinib, **B** The overall structure of the FLT3 kinase domain bound to Gilteritinib (PDB: 6JQR); the hinge region, juxtamembrane domain, and activation lobe are illustrated in black, **C** A detailed 3D representation of the interactions between FLT3 and Gilteritinib, **D** 2D structure of the FLT3 kinase domain bound to Gilteritinib

Regarding the binding mode of type I FLT3 inhibitors with FLT3 kinase domain, the co-crystalized structure of FLT3 and Gilteritinib (PDB: 6JQR) elucidates that Gilteritinib acts as ATP competitive agent. It interacts with active conformation (DFG-in) of the FLT3- TKD, forming two hydrogen bonds with Cys694 and Glu692 amino acids (Fig. 2) [49]. Furthermore, Gilteritinib has other hydrophobic interactions, specifically with the F691 gatekeeper residue. This illustrates that mutations within the gatekeeper TKD are among the main reasons for Gilteritinib’s resistance, which is enzymatically confirmed (Fig. 2) [50].

**Fig. 3:**
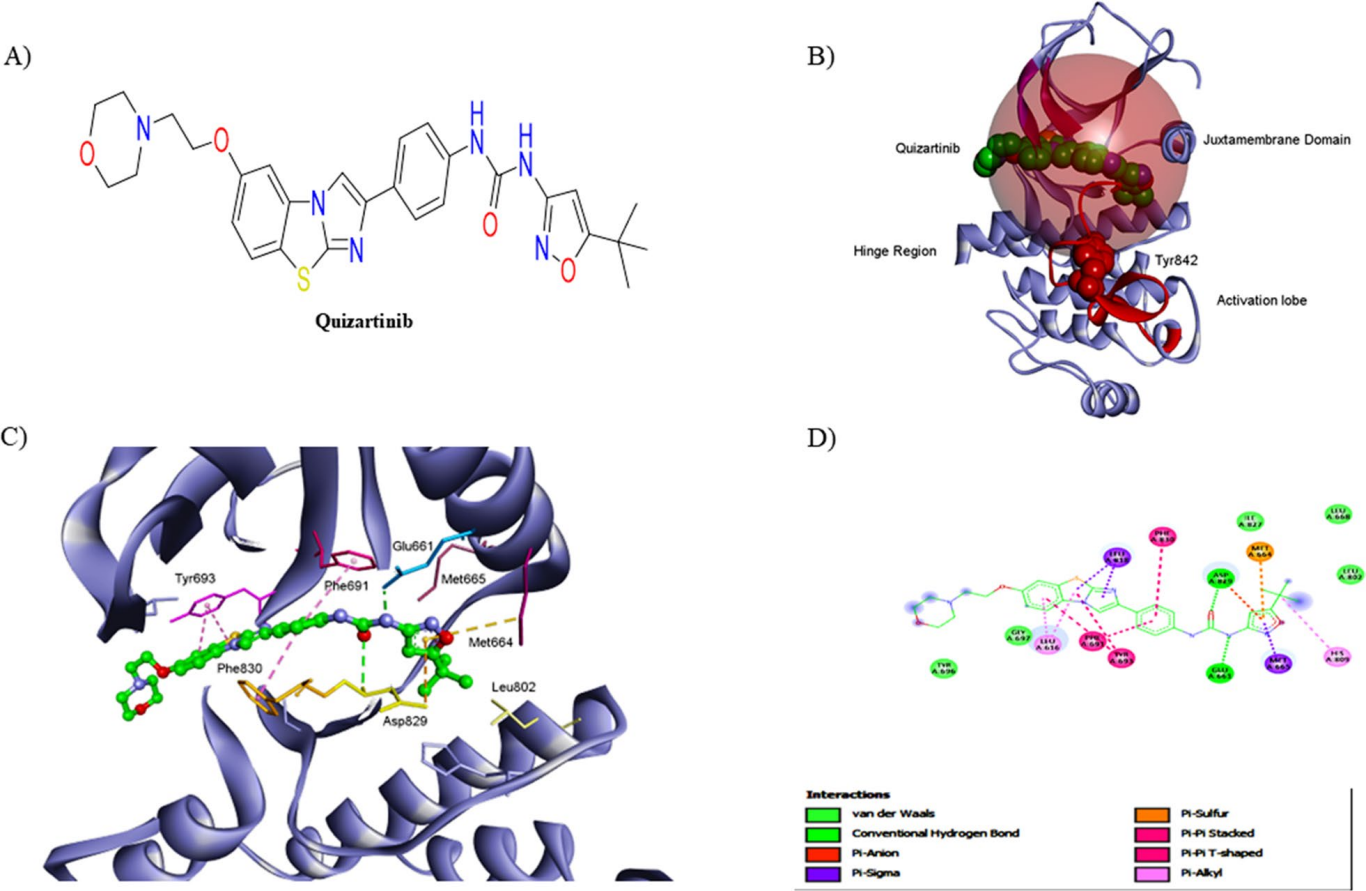
**A** The chemical structure of Quizartinib, **B** The overall structure of the FLT3 kinase domain bound to Quizartinib (PDB: 4XUF); the hinge region, juxtamembrane domain, and activation lobe are illustrated in black; **C** A detailed 3D representation of the interactions between FLT3 and Quizartinib, **D** 2D structure of the FLT3 kinase domain bound to Quizartinib

On the other hand, type II FLT3 hampering agent Quizartinib binds to the inactive conformation (DFG-out) of the TKD as a non-competitive ATP FLT3 inhibitor (PDB: 4XUF). It forms two main hydrogen bonds with Asp829 and Glu661 amino acids and many other hydrophobic interactions within the TKD (Fig. 3) [51]. Therefore, Quizartinib preserves its activity towards FLT3-ITD mutations within the juxtamembrane region but does not have the same activity towards FLT3- TKD mutations within the tyrosine kinase region (Fig. 3) [52].

So far, there are various indole-based FLT3 inhibitors, either FDA-approved or in clinical phases, such as Midostaurin, Lestaurtinib, Sunitinib, Crenolanib, etc. However, some obstacles stayed among these inhibitors, such as poor selectivity due to their multi-targets circumvention activity, FLT3 mutations, and some other drawbacks, which have resulted in a great battle for developing promising FLT3 inhibitors. Therefore, this review focuses on the indole and indole-based selective FLT3 inhibitors as promising scaffolds for developing more efficient FLT3 inhibitors, relying on the fact that FLT3 overexpression is involved in the aggressive and resistant AML pathophysiology.

## Indole based FLT3 inhibitors

Structure-based drug design represents a powerful strategy for affording more potent FLT3 inhibitors. In this respect, indole-based scaffolds had recently been widely used in developing multiple tyrosine kinase inhibitors due to their outstanding advantages, such as higher potency, selectivity, lower side effects, and so on [53]. Therefore, many indole-based FLT3 inhibitors were FDA-approved, such as Midostaurin, Lestaurtinib, etc. Midostaurin (**1**) is a semi-synthetic indole-based multiple-kinase inhibitor [54]. It had been primarily used as a protein kinase C inhibitor (PKCI). Then, it was known for its inhibitory activity versus many members of the tyrosine kinase class III family, namely VEGFR, PDGFR, c-KIT, and FLT3 [54] (Table 1**)**. Mechanistically; Midostaurin has a type I mode of binding with FLT3 receptor [13]. Phase IB clinical study revealed that the use of Midostaurin with chemotherapy (a cycle of induction with Daunorubicin 60 mg/m^2^ intravenously (IV) on days 1–3 and Cytarabine 200 mg/m^2^ by continuous intravenous infusion (CIV) on days 1–7) might improve the outcomes for newly diagnosed younger patients with FLT3-mutant AML [55]. Midostaurin was first studied in about thirty-two patients with advanced solid cancers in a phase I trial receiving doses of 12.5–300 mg daily in 28-day cycles [56]. This research protocol revealed that a dose of 150 mg per day or lower was well-tolerated. The most common toxicity signs were nausea, vomiting, diarrhea, and fatigue. Also, lymphocyte and monocyte levels were fundamentally decreased in patients who received ≥ 100 mg per day, which elucidated that Midostaurin impaired myeloid and lymphoid hematopoietic cascades and it could powerfully be suitable in some types of hematological malignancy [56]. Midostaurin was able to trigger G1 arrest and apoptosis in Ba/F3 cell lines with mutated FLT3 types with an IC_50_ value of 10 nM [46, 57]. Furthermore, Midostaurin was reported to hamper leukemia development in BALB/c mice with FLT3-ITD- leukemia [46]. Then, in phase IIB clinical trials, about twenty cases with mutated FLT3 with reverted AML or highly aggressive myeloblastic conditions were investigated versus individual Midostaurin therapy [58]. Then, the clinical outcome was detected in 70% of cases, where about 42% of cases with FLT3-wt and 71% with mutated FLT3 exhibited a clinical outcome, illustrating the anti-leukemic activity of Midostaurin in acute myeloid leukemia cases, specifically those with mutated FLT3 [59]. In RATIFY phase III clinical study, when Midostaurin was used in a combination with typically used chemotherapy (a cycle of induction with Daunorubicin 60 mg/m^2^ intravenously (IV) on days 1–3 and Cytarabine 200 mg/m^2^ by continuous intravenous infusion (CIV) on days 1–7), it enhanced both the overall and event-free human cases’ survivals. This research study recommended the usage of Midostaurin as a constituent in treating human cases at earlier ages [60]. In another phase III RATIFY clinical trial, more than 3200 patients aged nineteen to sixty were examined for mutated FLT3 AML (NCT00651261), which reported 717 suitable cases for this study [60]. The mean values of overall survival (OS) and event-free survival (EFS) for AML cases receiving Midostaurin were 74.7 and 8.2 months, whereas the OS and EFS values for the placebo group were 25.6 and 3.0 months [61, 62]. These outcomes from this RATIFY study resulted in the approval of Midostaurin in adults with newly diagnosed AML with FLT3 mutation in the United States, European Union, and other countries [61, 62]. However, investigated AML cases that relapse on chemotherapy in addition to Midostaurin in their treatment protocols; may still respond to other newer inhibitors, illustrating that Midostaurin's efficacy may be due to hampering other targets than FLT3 and that several Midostaurin resistance mechanisms present of which FLT3 mutations are not of the central authority [13].

**Table 1:** Indole-based FLT3 inhibitors

Compound name and its structure	Description		Refs.
	Target	FLT3, PKC, Syk, FLT-1, AKT, KIT, PDGFR-ẞ, and VEGFDR1/2	[47, 55, 58–60]
	Binding mode to FLT3 kinase	Type I	
	Mutation point	N676K/S/D, A627T/P, F691L, and G697R/S	
	In vitro targeted AML cells (IC_50_)	1–10 nM (in Ba/F3 cells expressing FLT3-D835V/D835del/ I836del/D853H/D835A/ D835E/D835Y/ D835N), and 4.14 nM (in Ba/F3 expressing FLT3-ITD)	
	Target	FLT3, TrKA, JAK2, and VEGFR	[63–67]
	Binding mode to FLT3 kinase	Type I	
	Mutation point	A627P	
	FLT3 Potency (IC_50_)	2.9, 11.9, and 3 nM (FLT3-ITD (in AML monocytes), FLT3-wt (in AML monocytes), FLT3-ITD (in Ba/F3 cells), respectively)	
	In vitro targeted AML cells (IC_50_)	5 nM (in Ba/F3 cells expressing FLT3-ITD)	
	Target	Dual FLT3, and MnK2	[69]
	Binding mode to FLT3 kinase	Type I	
	FLT3 Potency (IC_50_)	0.34 μM	
	In vitro targeted AML cells (IC_50_)	0.6 μM	
	Target	FLT3-ITD-F691L	[70]
	Binding mode to FLT3 kinase	Type I	
	FLT3 Potency (IC_50_)	114 nM	
	In vitro targeted AML cells (IC_50_)	0.34 μM	
	Target	FLT3	[71]
	Binding mode to FLT3 kinase	Type II	
	FLT3 Potency (IC_50_)	5.5 μM	
	In vitro targeted AML cells (IC_50_)	1.4–5.7 μM	

Lestaurtinib (**2**) is another indole-based kinase restrainer with a wide spectrum of inhibition. Like Midostaurin, Lestaurtinib had a great structure similarity to Staurosporine (Table 1**)**. It had an efficient inhibitory activity versus JAK2, FLT3, and TrkA with IC_50_ values of 0.9 nM, 3 nM, and lower than 20 nM, respectively [63]. Lestaurtinib displayed a strong circumvention ability against wild and mutated FLT3 in vitro via type I binding mode with FLT3 receptor [13], and it delayed mortality in xenograft studies [64]. Moreover, it was found that Lestaurtinib as a monotherapy resulted in a lessening of eruptions in both marginal blood tissues and bone marrow in about 36% of AML cases with mutated FLT3 in phase I/II clinical trials [65]. Later, a phase II clinical study was carried out, elucidating that the clinical therapeutic outcome was detected in AML cases regardless of the presence or absence of FLT3 mutation [44]. These clinical responses to Lestaurtinib and chemotherapy were evaluated in an indiscriminate phase III clinical study [66]. Regrettably, no observable variation in the light of comprehensive reduction or total endurance was noticed among cases exposed to chemotherapy individually (for 1–6 months, obtaining MEC, consisting of mitoxantrone 8 mg/m^2^ per day, etoposide 100 mg/m^2^, and Cytarabine 1000 mg/m^2^ per day all by I.V injection on days 1–5) or who followed by Lestaurtinib therapy. This illustrated that the use of Lestaurtinib after chemotherapy was inefficient for mutated FLT3 AML cases, which may be attributed to the pharmacokinetic properties of this scaffold. Moreover, another clinical study of Lestaurtinib in combination with chemotherapy (either Mitozantrone, Etoposide, Cytarabine [MEC], or high-dose Cytarabine [AraC]), combining Lestaurtinib with rigorous chemotherapy appeared possible for young-aged patients with a recently diagnosed FLT3-mutated AML, but actually, no observable clinical outcome was obtained; however, the clinical cases with higher than prolonged sustained 85% FLT3 inhibition percentage had enhanced the existence of those treated patients, proposing that prolonged FLT3 circumvention is vital [67]. Unfortunately, Lestaurtinib-treated AML cases with plasma levels exceeding 20 μM suffered from toxicity. Consequently, a low margin of safety, besides poor efficacy outcomes obtained from clinical trials, resulted in stopping further clinical development of Lestaurtinib [68].

In 2011, Diab et al. reported compound **3** as a dual FLT3 and Mnk inhibitor with K_i_ values of 0.34 μM and 0.11 μM, respectively **(**Table 1**)**. Consequently, this could be considered a great help to combat resistance mechanisms of acute myeloid leukemia cells as the inhibition of both FLT3 and Mnk2 resulted in increasing the apoptotic cell death of MV4-11 cells compared to inhibition of FLT3 or Mnk2 individually [69]. The cell viability assay revealed that compound **3** afforded highly promising antiproliferative activity versus MV4-11 cells with GI_50_ value lower than 1 μM upon 72 h cytotoxicity. Annexin V/PI and western blotting analyses depicted that the apoptotic effects and kinases’ inhibition of compound **3** versus the MV4-11 cells were displayed as dose- and time-dependent. After 72 h of exposure to MV4-11 cells, compound **3** triggered additional 14% apoptotic cells, and compound **3** could reduce the levels of p-STAT5 (Y694) and p-eIF4E (S209), proving its dual circumvention of FLT3 and Mnk2. Consequently, to evaluate the consequences of this dual inhibition of FLT3 and Mnk2 on the cell cycle progress, MV4-11 cells were treated with compound **3** for 48 h and examined by flow cytometry. Compound **3** produced superior modifications on cell distribution with 73, 74, and 82% DNA content in the G1/sub-G1, respectively, in a dose-dependent manner. Furthermore, a dose-dependent regression of pro-survival Mcl-1 protein (anti-apoptotic protein) was detected as a strong indication sign for inducing apoptosis in MV4-11 cells. Compound **3** specifically decreased levels of Mcl-1 and PARP anti-apoptotic proteins, illustrating the extra efficacy of compound **3** on these proteins as a dual FLT3 /Mnk2 inhibitor.

**Fig. 4:**
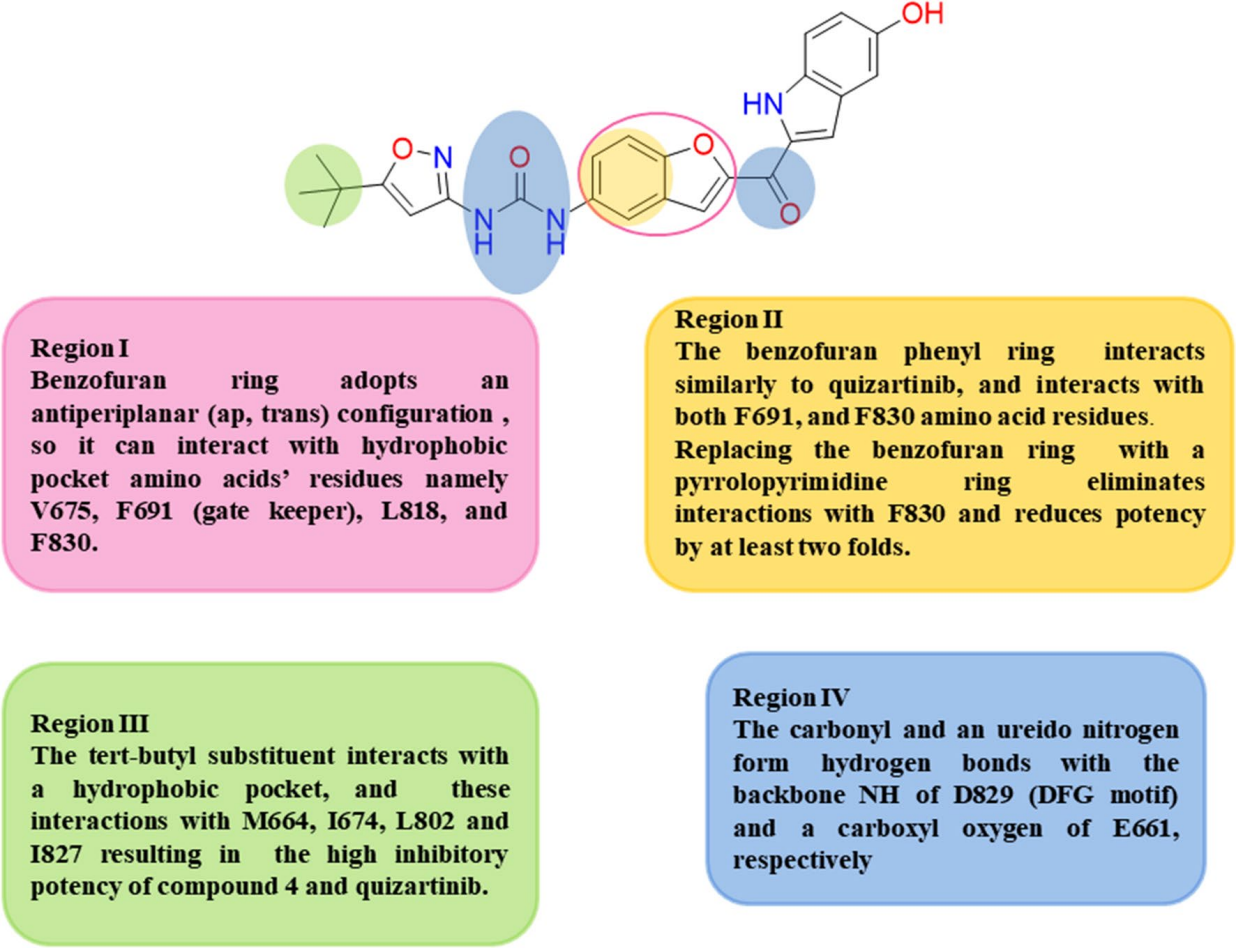
Hybrid mechanism and possible binding sites of compound **4** with the DFG-out model of FLT3 (PDB: 4RT7)

In 2020, Sellmer et al. synthesized a methanone indole derivative, compound **4**. It was considered a landmark achievement in the history of FLT3 inhibitors due to its specificity and selectivity **(**Table 1**)** [70], it showed a higher binding interaction tendency with FLT3-ITD-F691L (a derived drug-impedance gatekeeper mutator) with binding affinity (Kd) value equal to 114 ± 14 nM compared to Quizartinib; which has a Kd value of 340 ± 60 nM. The in silico studies revealed that compound **4** could be docked into both the DFG-in (PDB 4XUF) and the DFG-out (PDB 4RT7) conformations of the FLT3 binding site** (**Table 1**)**. At the same time, the bis-indolyl-methanone and the tert-butyl isoxazole core moieties of Quizartinib only can fit within the specific hydrophobic backward-pocket present in both cocrystallized biostructures of the FLT3. Within this context, compound **4** can be considered a type II tyrosine kinase inhibitor owing to its binding and stabilizing tendency with the inactive DFG-out FLT3 confirmation in the same manner as Quizartinib. Additionally, it was found that compound **4** has a higher affinity to bind to the mutated ITD FLT3 rather than the wild FLT3; however, Quizartinib can bind to both wild and mutated ITD FLT3 with similar affinity. Briefly, the benzofuran core scaffold of compound **4** adopts an antiperiplanar (ap, trans) configuration that allows repulsion prevention between the oxygen atoms; so this conformation permits the lipophilic benzofuran substituent to interact with a hydrophobic backward pocket involving V675, F691 (gatekeeper), L818, and F830 (DFG motif) **(**Fig. 4**)**. The benzofuran core scaffold’s phenyl ring overlays with the centric phenyl linker of Quizartinib and interacts in the same manner with both F691 from a certain edge and F830 from the other edge. The higher potency of compound **4** towards the D835Y mutant notably allows preservation of the activation loop in prolonged conformity that is favorable for compound **4** interaction and unfavorable for the interaction with Quizartinib and any other type II tyrosine kinase inhibitor regardless of the structural resemblance of compound **4** with Quizartinib. This could be attributed to being stabilized in a specific active-like conformation. Favorably, compound **4** displayed cytotoxic activity towards about nineteen hematopathological cell lines with a mean IC_50_ value equal to 0.34 μM and a potential selectivity versus three highly susceptible cell lines, MOLM-13, EOL-1, and MV4-11, with highly potent IC_50_ values of lower than 0.001 μM (Table 1**)**. In proteomics assay, it was found that among 249 tested well-known kinases additional to FLT3, only RET and ZAK/MLTK are predominantly sensitive to compound **4** with EC_50_ values equal to 443 nM, 277 nM, 1069 nM, respectively**.** Also, through flow cytometry AnnexinV/PI staining assay using MV4-11 cells, it was found that using compound **4** in concentrations 20 nM and 40 nM, respectively through 48 h incubation period, resulted in 25% of MV4-11 cells entered in apoptosis stage raised to 40% entered in late stages of apoptosis and necrosis in both doses. Herein, compound **4** can be considered a milestone that promotes further lead optimization to elucidate more specific, selective FLT3 inhibitors for optimum AML therapy.

In 2021, compound **5** was isolated as kimchi's most predominant indole alkaloid via the fermentation of the phytopathogenic fungus Sclerotinia sclerotiorum [71]. Compound **5** was reported as a potent and selective FLT3 inhibitor with an IC_50_ value of 5.5 μM **(**Table 1**)**. Specifically, compound **5** has an ATP-competitive FLT3 binding mode. Molecular simulation studies revealed that compound **5** binds preferentially to the FLT3 pocket in the DFG-out inactive conformation (PBD ID: 5X02) via forming several hydrophobic interactions with Leu612, Leu818, Val624, and Phe830 amino acid residues; compound **5** binds with DFG-PHE residue (PHE830) via π − σ interaction and one hydrogen interaction between compound **5** NH group and the main back skeleton oxygen atom of DFG-Asp residue (ASP829); consequently, compound **5** circumvention of the FLT3 activity via the adoption of type II binding mode. Cell culture and flow cytometric techniques showed that compound **5** circumvented various AML cells with IC_50_ value oscillating between 1.4 and 5.7 μM **(**Table 1**)** and highly promising cytotoxicity against different types of AML cells in a dose and time-dependent manner. In relation, compound **5** apoptotic activity is accompanied by dose-dependent reduction of the anti-apoptotic protein Bcl-2 scale and elevation of other apoptotic protein markers, including BAX, cleaved-PARP, and caspase3. Furthermore, western blotting analyses elucidated that the FLT3 autophosphorylation in MV4-11 and MOLM-13 cells was hampered by compound **5** at 5 and 10 μM, respectively, subsequently resulting in the attenuation of some down-stream signaling pathways of FLT3 phosphorylation, including MAPK, STAT5, ERK, and PI3K/AKT pathways**.** Lactate dehydrogenase (LDH) is an intracellular cytoplasmic enzyme, and the presence of LDH extracellularly is considered an unfailing cell death indication. Upon conducting an LDH assay, compound **5** intensifies the amount of released LDH; however, it affords a neglectable effect on the proliferation of both HUVEC cells (human immortalized cells) with IC_50_ value higher than 39.5 μM and 293 T-cells with IC_50_ value more than 52.7 μM) **(**Table 1**)**. As a result, compound **5** has selective cytotoxicity on the FLT3-expressing leukemia cells over normal cells. Eventually, upon conducting a cell viability assay, compound **5** attenuated the tumor proliferation rate and improved the antiproliferation effect of Sorafenib on the human MV4-11 cells transplanted in mice with significant inhibition of the FLT3 and its downstream signaling in line with previously mentioned in vitro results. Besides, the PARP cleavage assay presented that using a combination of compound **5** and Sorafenib exhibited the bottommost cell proliferation indicator (Ki67-positive) accompanied by the utmost noticeable cell apoptosis compared with those treated with compound **5** or Sorafenib alone. Thus, compound **5** represents a promising scaffold for further lead optimization for affording novel selective FLT3 inhibitors for the optimum treatment of AML.

Many limitations stand behind the wide application and further clinical development of the FDA-approved indole-based FLT3 inhibitors (Midostaurin and Lestaurtinib). First of all, low efficacy as a result of multiple resistance mechanisms specifically off-target mutations mainly decreased the potency of these inhibitors against FLT3 overexpressing AML cells, and this made these inhibitors potent only when included within combinatorial therapy regimens with other anticancer agents (such as Doxorubicin, Cytarabine, and Anthracycline). Secondly, poor specificity and selectivity towards FLT3 resulted in many off-target side effects. Eventually, lower eligibility for these inhibitors when they were included within the longstanding treatment protocols because of the rapid emergence of resistance versus those agents and their low toxicity margins, making them less tolerated for AML patients (specifically, Lestaurtinib).

Compounds **3**, **4,** and **5** were not fully studied regarding their physicochemical properties, their safety profiles through the in vivo studies, and the consequences of long-term treatment with these biomolecules on FLT3 AML patients. Although, their anti-apoptotic activities were detected. Therefore, in the future, those indole-based FLT3 inhibitors (compounds **3**, **4,** and **5**) should be fully investigated with all their proposed pharmacokinetic and pharmacodynamic properties to facilitate the development of more potent FLT3 inhibitors.

## Azaindole based FLT3 inhibitors

In the continuation for searching more potent and selective FLT3 inhibitors, many heterocycles have great structure similarity with the indole ring; they are included in various new biomolecules used as FLT3 inhibitors such as azaindole ring [72]. An interesting study conducted by Ganser et al. reported compound **6** as a tris kinase inhibitory agent versus FLT-3, GSK-3β, and VEGFR-2 with residual activity percentages of 0, 2, 1% with IC_50_ values of 18, 9, and 48 nM, respectively upon using 10 μM as compound **6** dosage **(**Table 2**)** [73]. Based on MTT assay results, it was found that the three days remedy with the azaindole derivative compound **6** showed cell viability percentage versus the HT-29 (human colon adenocarcinoma cells), and, Molm-14 about 4.4% with 52 μM as compound **6** dosage and 2.40% with 50 μM as compound **6** dosage**,** respectively; and over 5 days remedy against Mkn-45 (gastric cancer cells) of 68.58% with 50 μM as compound **6** dosage. Additionally, the proapoptotic efficacy of compound **6** versus HUVECs (human umbilical vein endothelial cells) and Mkn-45 (gastric cancer cells) was evaluated, affording apoptosis-inducing percentage of 66.3% with 2.6 μM as compound **6** dosage, and 28.3%, with 20 μM as compound **6** dosage, respectively. Thus, the authors postulated compound **6** represented an important milestone for affording more potent FLT3 inhibitors because of its great selectivity and potency towards Molm-14 AML FLT3 expressing cells.

**Table 2:** Azaindole-based FLT3 inhibitors

Compound name and its structure	Description		Refs.
	Target	FLT-3, GSK-3β, and VEGFR-2	[73]
	Protein kinases (IC_50_)	18, 9, and 48 nM, respectively	
	In vitro cell viability percentage	4.4% at a concentration of 52 μM, 2.40% at a concentration of 50 μM (in HT-29 (human colon adenocarcinoma cells), and, Molm-14 AML cells, respectively)	
	Target	FLT3-ITD, CSF1R, and KIT	[74, 75]
	Binding mode to FLT3 kinase	Type II	
	Protein kinases’ Potency (IC_50_)	11, 13, and 27 nM/L, respectively	
	Target	FLT3	[72]
	Binding mode to FLT3 kinase	Type II	
	FLT3 Potency (pIC_50_)	8.02	
	Target	FLT3	[72]
	Binding mode to FLT3 kinase	Type I and II	
	FLT3 Potency (pIC_50_)	9.49	

In 2014, smith et al. reported compound** 7** (containing a central pyridine ring alternative to phenyl) and a methylamine linker (alternative to amide or urea linkers in another type II kinase hampering agents) as a new FLT3 hampering agent with experimental inhibitory activity versus the Quizartinib impedance FLT3 F691L mutation [74, 75]. Interestingly, western blotting assay presented that compound **7** has a tris kinase inhibitory activity against CSF1R (colony-stimulating factor 1 (CSF-1), which is responsible for the enrollment of colony-stimulating factor-1 receptor (CSF-1R) stating macrophages that synthesize the prime cell kind through these gigantic tumor cells with IC_50_ equals to 13 nmol/L, KIT (c-Kit receptor tyrosine kinase) with IC_50_ value of 27 nmol/L, and FLT3-ITD with IC_50_ equals to 11 nmol/L **(**Table 2**)**. In vitro experiments utilized recombinant proteins' technology showed that compound** 7** has a favored affinity to FLT3-ITD with IC_50_ value of 10 nmol/L relative to the autoinhibited innate wild FLT3 with IC_50_ value of 0.4 μmol/L **(**Table 2**)**. Computational studies upon compound** 7** preserve its activity contrary to the F691L-FLT3 mutant similar to its costructure binding mode with CSF1R (PDB:4R7H), which clarifies that compound** 7** binding interaction is negligibly affected by mutation of F691L gatekeeper kinase residues. This type of mutation allows entrance to the allosteric binding site, which is end-to-end to the ATP pocket highly related to TKI resistance; therefore, compound** 7** is considered a type II kinase inhibitor. Interestingly, Immuno blotting assay elucidated that compound** 7** circumvents FLT3 signaling cascades in FLT3-ITD-MV4-11 cells with a biological IC_50_ value of 18 nmol/L; however, it has noticeably lower efficacy towards RS4-11 harboring cells containing only innate wt-FLT3- with biological IC_50_ value of 1.8 μmol/L, illustrating compound** 7** tendency towards the FLT3-ITD-mutant type. Furthermore, via carrying the antiproliferation studies of compound** 7** and Quizartinib on FLT3-ITD, FLT3-ITD/F691L, and FLT3-ITD/D835Y Ba/F3 harboring cells, compound** 7** affords IC_50_ values of 0.13, 0.097, and 4.7 μM, respectively, contrary to Quizartinib, which can hamper both innate wild and mutant FLT3 phenotypes’ proliferation. Similarly, compound **7** can inhibit the propagation of Molm14 and F691L -Molm14 with IC_50_ values of 0.35 and 0.67 μM, respectively. The anti–FLT3-ITD affinity of compound** 7** was assured via establishing the in vivo MV4-11 xenograft model where compound** 7** is given a 30 mg/kg dosage, resulting in significant tumor growth suppression. On one side, compound** 7** affords similar attenuation of autophosphorylation and down-proceeding signaling of FLT3 in both MOLM14 F691L and hμMan plasma parental MOLM14 cells; therefore, higher obtained plasma levels of compound** 7** will have advanced clinical potency in either clinic visitors with innate FLT3-ITD or those who obtained the FLT3-ITD-F691L as a result of the previous remedy with Quizartinib or Sorafenib. Conversely, Quizartinib affords diminished activity versus F691L -MOLM14 cells compared with intravenously injected MOLM14 cells in the same assay. Critically, it was found that the absence of F691L mutations as a clinical impedance technique for compound** 7** in about nine tested patients in this study; powerfully clarifies the diminishing efficacy of compound **7** in like this mutation.

**Fig. 5:**
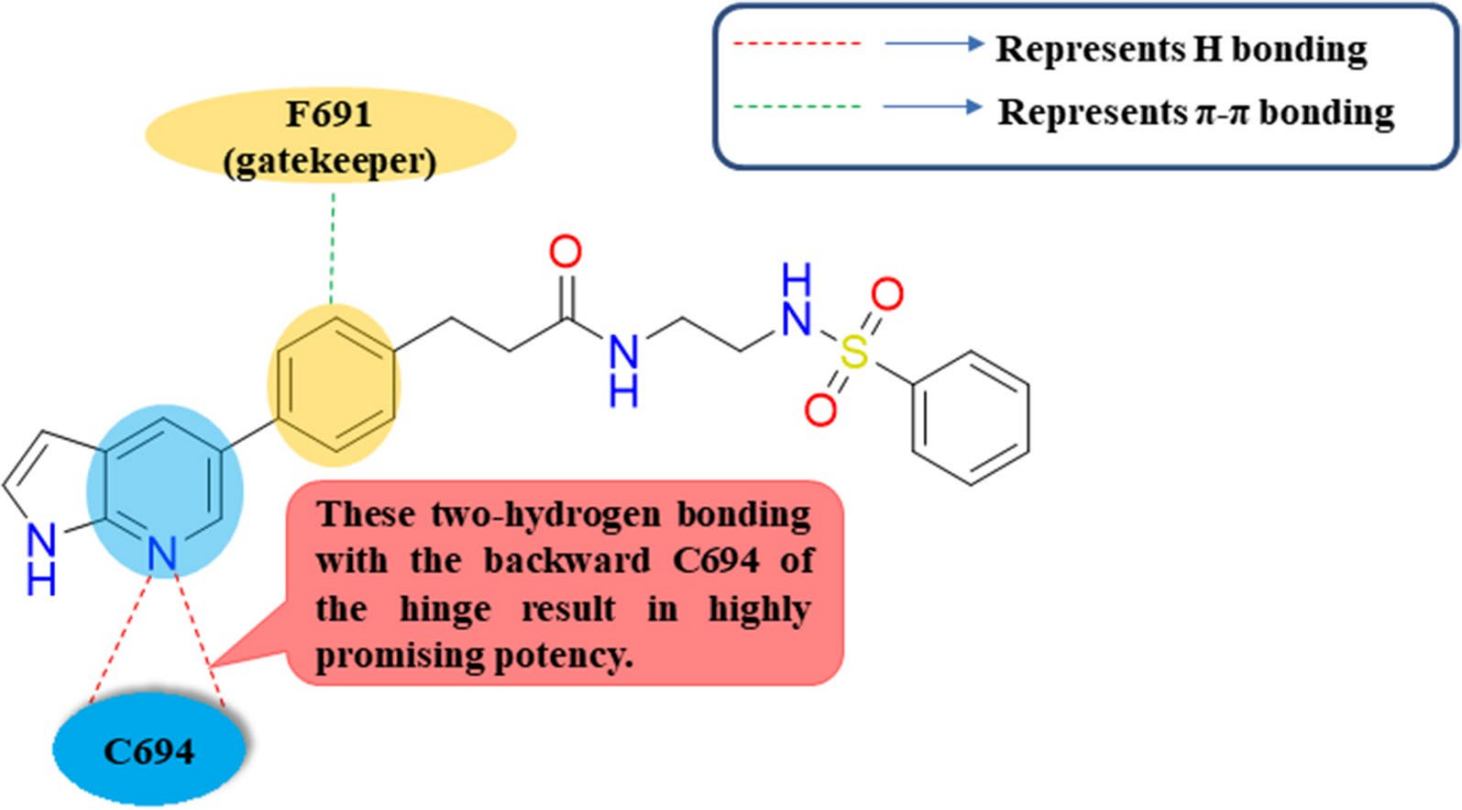
Simple 2 D diagram of the predicted binding modes of compound** 9** into DFG-out FLT3 kinase homology (PDB ID: 4RT7); illustrating two main hydrogen bonding interactions of compound** 9** with Cys694 and one π-π stacking interaction with F691 in the DFG-out model

In continuation of searching about further FLT3 suppressors, Grimm et al. in 2019 repositioned compound **8** with a pIC_50_ value of 8.02 ± 0.05 relative to the hampering capability of the reference Quizartinib inhibitor with a pIC_50_ value of 8.30 ± 0.07 as a brilliant initial start to ameliorate novel FLT3 inhibitors instead of traditional non-selective FLT3 inhibitors [72]. Among the synthesized FLT3 inhibitors in this study, compound **9** was the greatest FLT3 inhibitor with a pIC_50_ value of 9.49 ± 0.08 **(**Table 2**)**. Besides, compound **9** showed fortunate Physico-chemical properties represented with lipophilic efficiency (LipE) value of 6.5 [72]. Upon stopping on computational chemistry CADD results, it was concluded that both compound **8** and compound **9** fit into the FLT3 DFG-out cocrystallized structure with Quizartinib (**PDB 4RT7**); Compound **8**’s pyridine ring forms one hydrogen bonding with the backward C694 of the hinge and the neighboring phenyl ring afforded π-interaction with gatekeeper F691with no observed isoquinoline interactions with the FLT3 hinge; in the same manner as the docking interactions of **9** with an extra hydrogen-bond with C694, which probably results in highly promising potency **(**Fig. 5**)**. Additionally, compound **9** has another overlapped binding form with the FLT3 DFG-in structure (PDB: 3FMD) relative to other kinase inhibitors consequent from the phenotypical kinase inhibitor **H-89** that inversely circumvented the ATP binding with the preserved binding region of cAMP-dependent protein kinase (PKA) as PKA inhibitor.

Regarding azaindole-based scaffolds (**6** and **7**), each acted as a tris kinase inhibitor (FLT-3, GSK-3β, VEGFR-2, and CSF1R, KIT) and FLT3-ITD, respectively), so, they could combat against both on and off-target mutations related to FLT3 AML harboring cells. Therefore, they showed highly potent cytotoxicity versus different FLT3 AML cell lines (such as Molm14 and F691L -Molm14); however, no cytotoxicity assay was carried out for compound **9**. Lack of study of physicochemical properties and in vivo properties such as safety profile, anti-apoptotic activity, and effect of long-term treatment on FLT3 AML cases; resulted in a poor application and hampered additional investigations and improvements for those azaindole-based FLT3 inhibitors.

## Oxindole based FLT3 inhibitors

Drug repositioning stands among the best approaches that might rapidly produce promising FLT3 inhibitors [76]. Oxindoles, a class of heterocyclic compounds, displayed fundamental biological activities, including anticancer activity [77]. For instance, Sunitinib (**10**) is an orally ingested oxindole-based small-structured tyrosine kinase inhibitor (TKI) that was officially permitted for the remediation of renal cell carcinoma, gastrointestinal stromal, and pancreatic neuroendocrine tumors (Table 3**)** [78]. It inhibited majorly versatile tyrosine kinases such as FLT3, c-KIT, PDGFR, and VEGFR via exhibiting the type I binding mode with the FLT3 receptor [13]. In many in vivo xenografted studies, Sunitinib displayed inverse leukemic activity in numerous mice and bone marrow transplanted models [79]. There are two stages of phase I in clinical trials for Sunitinib as monotherapy in AML cases were done [80]. These two studies detected clinical outcomes such as a short period of partial retardation. After phase I studies, one set of these previously tested cases commenced a phase II research work to assess Sunitinib usage in combination with traditionally used chemotherapy (Cytosine arabinoside (Ara-C)/Daunorubicin induction (7:3) followed by three cycles of intermediate-dose Ara-C) in FLT3-mutated AML cases higher than six decades in age. The percent of total diminution was about 59% in wholly tested cases, 53% in FLT3-ITD cases, and 71% in cases with mutated FLT3-TKD types [41]. This proposes the efficacy of combinatorial therapy in AML cases, and additional research studies, including more cases, are ensured. In phase I/II study of Sunitinib in conjunction with induction and consolidation chemotherapy, fifty percent of patients with FLT3- ITD and thirty-eight percent with FLT3-TKD obtained complete alleviation [80]. During this research study, dose-toxicity profiles were tested for three patients, which presupposed dose reduction. So, Sunitinib was not approved for AML or other mutated FLT3-expressing cancer types [80].

**Table 3:** Oxindole-based FLT3 inhibitors

Compound name and its structure	Description		Refs.
	Target	FLT3, c- KIT, PDGFR-ẞ, and VEGFDR2	[35, 40, 67, 69, 78–80]
	Binding mode to FLT3 kinase	Type I	
	Mutation point	A848P, A627P, F691L, and Y842C	
	FLT3 Potency (IC_50_)	50, and 250 nM (FLT3-ITD (in MV4-16 cells), and FLT3-wt (in RS4-16 cells), respectively)	
	In vitro targeted AML cells (IC_50_)	8 and 15 nM (in MV4-16 expressing FLT3-ITD, and OC1-AML5 cells expressing FLT3-wt, respectively)	
	Target	FLT3-wt, FLT3-D835Y, and FLT3-D835H	[81]
	Binding mode to FLT3 kinase	Type I	
	FLT3 Potency (IC_50_)	40, 8, and 4 nM, respectively	
	In vitro targeted AML cells (IC_50_)	40 nM (HCD-57; murine erythropoietin dependent erythroleukemia cells) expressing FLT3-ITD, FLT3- D835Y, and FLT3-D835H)	
	Target	FLT3, FLT3-ITD, and FLT3-D835Y	[82]
	Binding mode to FLT3 kinase	Type I	
	FLT3 Potency (IC_50_)	5.3, 0.7, and 2.7 nM, respectively	
	In vitro targeted AML cells (IC_50_)	24.8, 5.9, and 5.5 nM (MV4-16, FLT-ITD- MV4-16, and MOLM-18, respectively)	
	Target	FLT3-ITD	[83]
	FLT3-ITD Potency (IC_50_)	1.25 μM and17.5 μM (with % inhibition 90.9, ≥ 100.0, respectively)	
	In vitro targeted AML cells (IC_50_)	2.2 μM (MV4-16)	
	Target	FLT3-ITD- D835H, FLT3-ITD- F691L, and FLT3-D835H	[84]
	Binding mode to FLT3 kinase	Type I	
	FLT3 Potency (IC_50_)	0.83, 1.5, and 1.3 nM	
	Target	wt-FLT3, FLT3-ITD, and FLT3/D835Y	[85]
	Binding mode to FLT3 kinase	Type I	
	FLT3 Potency (IC_50_)	0.87, 0.25, and 0.32 nM	
	In vitro targeted AML cells (IC_50_)	1.0 nM	
	Target	FLT3, FLT3-ITD, and FLT3-D835Y	[86]
	Binding mode to FLT3 kinase	Type II	
	FLT3 Potency (IC_50_)	1.8, 0.8, and 1.9 nM, respectively	
	In vitro targeted AML cells (IC_50_)	23.5 nM (FLT3-ITD- MV4-16), and 35.5 nM (MOLM-18)	
	Target	FLT3, CDK-2, EGFR, and VEGFR-2 (KDR)	[87]
	Binding mode to FLT3 kinase	Type II	
	FLT3 Potency (IC_50_)	0.546 µM	
	In vitro targeted cancer cells (IC_50_)	3.56 µM (MCF-7)	
	Target	FLT3, PDGFRα, VEGFR-2 (KDR), Aurora A and B	[88]
	Binding mode to FLT3 kinase	Type I	
	FLT3 Potency (IC_50_)	74.85, 24.15, 46.65, 16.83, and 29.38 nM, respectively	
	In vitro targeted AML cells (IC_50_)	6.84 and 2.97 µM (CCRF-CEM, and SR leukemia cells, respectively)	
	Target	FLT3-ITD	[89]
	FLT3 Potency (IC_50_)	2.49 μM	
	In vitro targeted AML cells (IC_50_)	4.3 and 8.7 μM (FLT-ITD- MV4-16, and FLT3-wt-THP-1cells, respectively)	

Chen et al. in 2016 reported compound **11**, primarily known as a JAK3 kinase inhibitor; however, compound **11** was 6 times more potent and selective than FLT3 [81]. Briefly, compound **11** exhibited powerful circumventing activities versus FLT3-WT, FLT3-D835Y, and FLT3-D835H with IC_50_ equal to about 40 nM, 8 nM, and 4 nM, respectively, but much-reduced efficacy versus JAK3 and c-Kit with IC_50_ equal to 250 nM and about 500 nM, respectively, besides compound **11** hampered the mutated FLT3-D835 more efficiently relative to FLT3 **(**Table 3**)**. Consequently, compound **11**, as a strong and specific FLT3 hampering agent, could circumvent FLT3-ITD- leukemia cell lines. Compound **11** suppressed the phosphorylation of both FLT3 and its down-proceeding signaling mediators, such as ERK and Akt, in both FLT3-ITD- and FLT3-D835Y- HCD-57 cells; however, Sorafenib displayed a fundamental circumvention on the FLT3-ITD cells rather than FLT3-D835Y mutated cell lines. Furthermore, based on the previous computational studies about the tyrosine kinases’ catalytic region, the D835 region mutation of FLT3 resulted in modification of the kinase region’s conformation. It preserved it in the DFG-in active motif, which is unreachable to Sorafenib as a type II inhibitor. Fortunately, in vitro and in vivo experiments’ results regarding compound **11** revealed that compound **11** fitted type I inhibitors owing to its efficient ability to interact with both DFG-in and DFG-out conformations of FLT3; this represented a marvelous step in affording inhibitors versus TKDs-FLT3 and ITD- FLT3 which are instantly required. Moreover, compound **11** showed potent antiproliferative activity versus transformed FLT3-ITD and D835 mutant cells; for instance, compound **11** inhibited the proliferation of HCD-57 (murine erythropoietin dependent erythroleukemia cells) expressing FLT3-ITD, FLT3- D835Y, and FLT3-D835H with IC_50_ equal to about 40 nM, while it demonstrated no efficacy versus the parent HCD-57 or JAK2V611F relative to Sorafenib that inhibited the proliferation of FLT3-ITD-HCD-57 cells rather than other tested cells depriving of FLT3 expression. Upon carrying out cell cycle arrest and flow cytometric assays, compound **11** meaningfully lessened G2- and S-phase cells and increased G1-phase cells in both FLT3-ITD and D835Y expressing HCD-57 cells with wider efficacy than Sorafenib in addition to the rise in percentage proportions of apoptotic and necrotic cells after compound **11** therapy, but Sorafenib displayed such this outcome on the FLT3-ITD cells only. To evaluate the in vivo FLT3 signaling cascades’ inhibitory activity of compound **11**, a xenograft model was prepared via I.V. injection of mice with FLT3-D835Y expressing HCD-57 cells. After the passage of 3 weeks later, mice are injected with a single dosage of compound **11** at 100 mg/kg and sacrificed 4 h later. Extraction of bone marrow and spleen cell antibodies versus phosphorylating agents, including FLT3, ERK1/2, and Akt, elucidates that compound **11** injection meaningfully circumvented FLT3 phosphorylation and its down-proceeding signal transmission.

One of the highly selective reported scaffolds against FLT3 was compound **12**, reported by Ma et al. in 2017 [82]. Notably, compound **12** was tested versus a series of 39 main carcinogenic human proteins at a dosage of 0.1 mM; surprisingly, compound **12** had remarkable inhibitory percentages against 4 kinases, namely FLT3, FLT3-ITD, FLT3-D835Y, and PDGFRa-T674I with values of 95, 105, 93, and 98%, respectively **(**Table 3**)**. In relation, compound **12** displayed higher hampering activity versus FLT3, and weaker activity versus both PDGFRa and c-Kit with IC_50_ values of 5.3, 114.6, and 227.0 nM, respectively **(**Table 3**)**, which can illustrate the greater potency and selectivity compound **12** versus FLT3. Moreover, compound **12** displayed higher catalytic domain circumvention of both mutant FLT3-ITD and FLT3-D835Y with IC_50_ equal to 0.7 and 2.7 nM, respectively; thus, compound **12** was about two thresholds sensitive towards mutated FLT3 than native FLT3 with IC_50_ value of 5.3 nM, which facilitates the use of this scaffold as a specific FLT3 inhibitor versus Quizartinib- FLT3-ITD- resistant mutated domain kinases. Besides, upon testing the FLT3 phosphorylation in MV4-11 cell lines, it was obvious that compound **12** can act as a direct inhibitor for FLT3 phosphorylation in a dose-dependent mode after 2-h therapy. Furthermore, a potent antiproliferative activity of compound **12** was exhibited against MV4-11 with IC_50_ equal to 24.8 nM compared to Sunitinib with IC_50_ equal to 38.5 nM. Compound **12** afforded a critical anticancer activity against mutated FLT-ITD- MV4-11 and MOLM-13 acute myeloid leukemia cells with IC_50_ values of 5.9 and 5.5 nM, respectively. In contrast, compound **12** showed less efficient antiproliferative activity versus FLT3-negative Jurkat and HL-60 cells. As a result, compound **12** had a powerful inhibitory and selectivity versus MV4-11 and MOLM-13 especially expressing mutated FLT3 acting as a direct FLT3 inhibitory agent. A xenograft model of a subcutaneous tumor MV4- 11 cell line in athymic nude mice was carried out to elucidate the antiproliferative activity of compound **12**, which displayed a low cancer progress inhibition proportion by 52.43% at the dose of 5 mg/kg twice orally per day, 88% at the dose 10 mg/kg twice orally per day, compared to Sunitinib with meaningfully circumvented tumor progress by 81.01% inhibition ratio at the dose 10 mg/kg orally twice per day. Compound **12** displayed robust hampering activity on tumor progress at 20 mg/kg and 40 mg/kg doses with tumor circumvention ratios of 99.78% and 100.00%, respectively.

Recently in 2018, Nemes et al. reported the phenyl methoxy ester Sunitinib derivative compound **13**, through western blotting assay, can exhibit fundamental inhibitory activity versus FLT3-ITD at a dosage of 1.25 μM compared with reference Sunitinib with inhibition percentages of 90.9%, and 92.3%, respectively **(**Table 3**)** [83]. Additionally, this compound displayed a promising antiproliferative activity against MV4-11 acute myeloid cells with IC_50_ equal to 2.2 μM. Moreover, compound **13** exhibited good lipophilic properties with a logP value of 4.73, in addition to the presence of monocarboxylic acid in this compound that may be reflected in a better oral bioavailability, carriers' conjugation, and biological activity of this scaffold.

In the way of searching for selective FLT3 inhibitors even with inhibitory activity versus other tyrosine kinases, Bensinger et al. in 2019 reported the discovery of a new scaffold **14** that showed greater specify towards FLT3 kinases subtypes upon testing of 100 nM as compound **14** dosage versus a series of 34 kinases including FLT3(ITD- D835H), FLT3(ITD- F691L), and FLT3(D835H) with binding affinity values of 0.83 nM, 1.5 nM, and 1.3 nM, respectively **(**Table 3**)** [84]. Compound **14** exhibited highly powerful circumvention proportions against FLT3 and FLT3(D835Y) of about 95% and 99%, respectively. Compound **14** displayed lower circumvention ratios than other kinases, including the PDGF (platelet-derived growth factor) receptor family comprising the DFG-1 cysteine domain, including c-KIT, PDGFRα, and PDGFRβ with values of 34%, 24%, and 14%, respectively. Noticeably, Sunitinib and Crenolanib showed a progressive tendency toward wild FLT3 and FLT3- ITD than compound **14**; even though Midostaurin is less effective, Crenolanib and Quizartinib missed the tendency toward both ITD and point-mutated FLT3; however, the tendency was amplified for compound **14**. Exclusively, compound **14** displayed about 14 thresholds higher affinity versus the drug-resistant FLT3(ITD, F691L) mutation relative to Crenolanib. Upon carrying molecular dynamics study for compound **14**, it was found that compound **14** co-structured with the FLT3-DFG-in homology form over 110 ps affords only lower RMSD values of 0.5 to 1.0 Å relative to the original ligand co-structure, estimating that the protein − ligand complex is extremely constant, which appears in the higher antiproliferative activity of compound **14** versus Sunitinib in MV4-11 cells. Regarding in silico studies for compound **14** on FLT3, the active FLT3 conformation model is designed as a new homology model utilizing c-KIT as a template (PDB 1PKG) with about 79% sequence similarity and 64% sequence identity because there was no reported crystal structure of FLT3 in the active DFG-in conformation. It was found that the ATP-binding domain of FLT3 owned three critical cysteine residues, including two of them, gatekeeper 3 Cys694 and gatekeeper 4 Cys695 amino acid residues, which are portions within the hinge region; however, Cys828amino acid residue presents within the DFG-motif. Potent kinases’ hampering agents should make either hydrogen bond donating or accepting interactions with the main skeleton moieties of the hinge region. Compound **14**’s top poses showed acceptable binding modes like Sunitinib. Besides, the presence of methyl group at position 4 adjacent to Cys694 affected the sulfonamide group's location, somewhat obliging a robust hydrogen bond donor and/or acceptor interaction with Lys644. Furthermore, the highly predominant antiproliferative activity of compound **14** was observed with an IC_50_ value of 6 nM compared to Sunitinib with an IC_50_ value of 54 nM, and type I inhibitors Midostaurin (about two thresholds), Crenolanib (about two thresholds), and HY (hypothemycin) (about two and a half thresholds) in MV4-11 cells. This scaffold also preserved its selectivity versus THP1 (wild FLT3), NOMO1, U937, and Jurkat cells (FLT3-negative). Also, compound **14** exhibited promising antiproliferative activity against FLT3-ITD-MOLM-14 cells with an IC_50_ value of 7.8 nM.

**Fig. 6:**
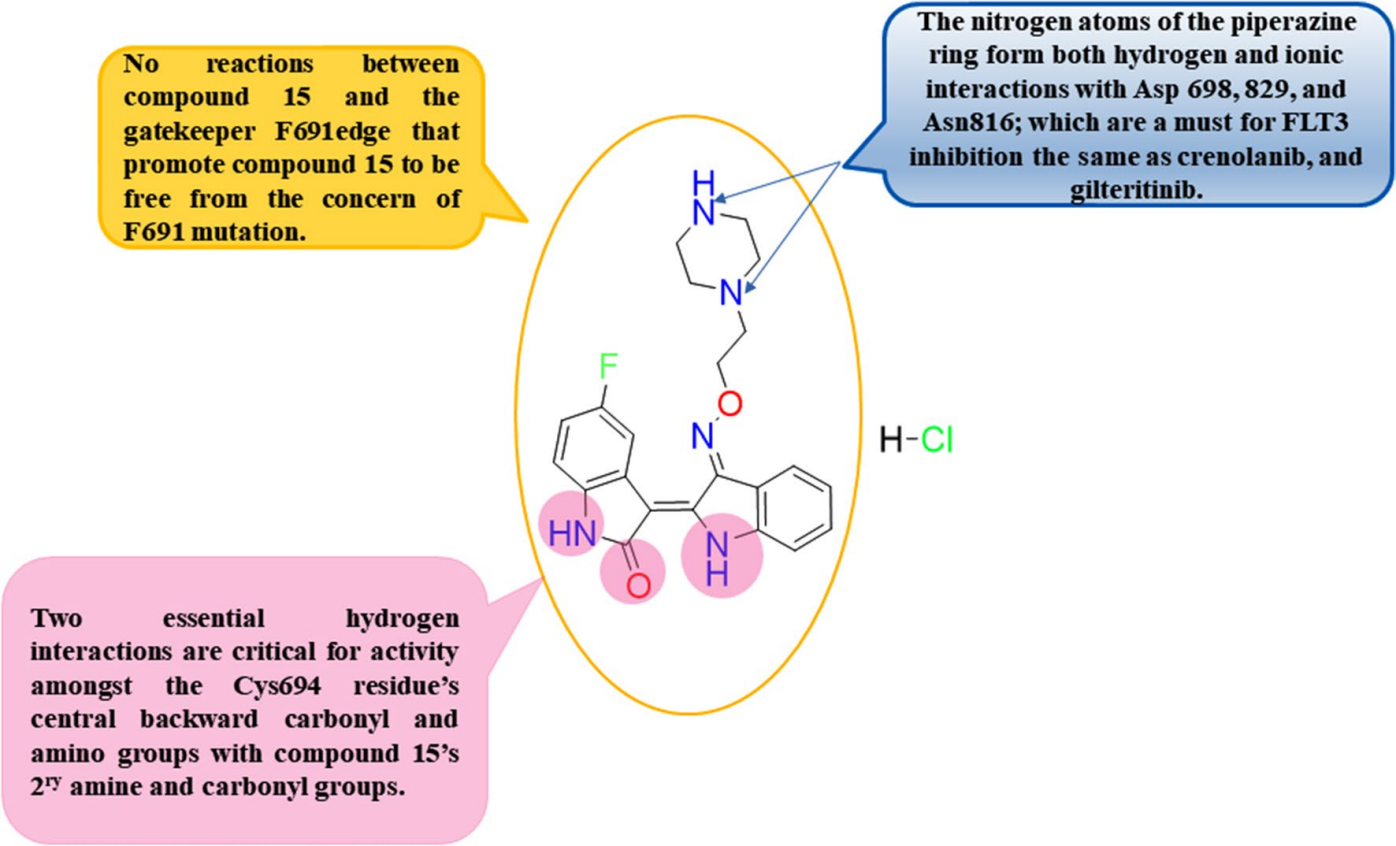
Structure–activity relationship of compound** 15**

An interesting study conducted by Jeong et al. in 2020 involved the synthesis of 3 substituted oxime indirubin-based scaffolds as strong type I FLT3 restraints designed to facilitate the therapy of AML [85]. Among all FLT3 inhibitors’ synthesized derivatives, compound **15,** through immune blotting analysis**,** showed higher circumvention against wt-FLT3 activity, affording IC_50_ value of 0.87 nM **(**Table 3**)**. Upon investigation of the kinase selectivity profile for compound **15**, it was detected that the hampering activity of compound **15** versus the mutant FLT3/D835Y with IC_50_ value of 0.32 nM converted to be extra powerful over versus the native kind of FLT3 with IC_50_ value of 0.87 nM. Moreover, compound **15** had a robust empathy towards FLT3-ITD, showing a binding affinity Kd value of lower than 0.25 nM, illustrating the higher circumvention affinity against FLT3 rather than CDKs’ inhibition (21, 42, and 54% circμMvention against CDK1, 2, and 5 at 100 nM dose, respectively), unlike known reported data about indirubin based scaffolds that are well known to be CDK inhibitors. Computer-aided investigations revealed compound **15** and the previously reported FLT3 hampering agents, Crenolanib and Gilteritinib, were fitted into the DFG-in motif FLT3 cocrystallized structure pattern (PDB ID: 3LCD**)** using the previously published method constructed by Yi-Yu Ke et al. Upon relying on docking studies’ results, compound **15** fitted effectively to the ATP pocket located within the hinge domain and the FLT3 phosphate-binding region where there are two essential hydrogen interactions amongst the Cys694 residue’s central backward carbonyl and amino groups with the indirubin-based compound **15**’s 2^ry^ amine and carbonyl groups **(**Fig. 6**)**. Surprisingly, no reactions between compound **15** and the gatekeeper F691 edge that can illustrate why compound **15** might be free from the worry of F691 mutation. In this computational study, the nitrogen moieties of compound **3**'s piperazine ring form both hydrogen and ionic interactions with Asp 698, 829, and Asn812 **(**Fig. 9**)**; this could exert a fundamental role for FLT3 circumvention ability where compound **15** revealed IC_50_ equals to 0.87 nM with the same occupying manner for the binding pocket like Crenolanib, and Gilteritinib (Table 3**)**. Notably, via cell viability assay, compound **15** showed a higher antineoplastic activity versus wild FLT3 expressing MV4-11 with GI_50_ worthing about 1.0 nM (Table 3**)**. Furthermore, compound **15** were tested versus various FLT3-ITD -AML cell lines including wild MOLM15, MOLM15-ITD, MOLM15-ITD-D835Y, and MOLM15-ITD-F691L cells, which represented clinically common mutated kinase expressed cells with FLT3 evolution dependency. Compound **15** afforded an identical dose-dependent antineoplastic activity against native kind of MOLM15 cells with GI_50_ value equal to 4.88 nM relative to Gilteritinib with GI_50_ worthing about 4.94 nM, and with a GI_50_ worthing about 1.85 nM versus MOLM15-ITD cells that indicates more sensitivity to MOLM15-ITD cells than native kind of MOLM15 with the same manner of Gilteritinib (Table 3**)**. In relation, compound **15** and Gilteritinib as type I hampering agent used member; preserved powerful antiproliferation efficacy against MOLM15-ITD-D835Y mutant cells with GI_50_ value of 1.87, and 1.91 nM, respectively; however, Quizartinib as type II kinase inhibiting agent revealed lower hampering activity with GI_50_ value of 6.72 nM. Moreover, regarding MOLM15-ITD-F691L cells, compound **15** and Gilteritinib afforded more potent antineoplastic activity with a GI_50_ value of 3.27 and 5.24 nM, respectively, over Quizartinib with GI_50_ value of 13.58 nM. In continuation, compound **15** and positive standard Gilteritinib were additionally tested for their circumvention of the down-proceeding FLT3 activation’s signal transmission pathway in MV4-11 cells with dosing of 1 μM, 100, and 10 nM. It was found that compound **15** showed an optimum obstruction of the Erk1/2 and STAT5 phosphorylation unfluctuating at the bottommost estimated dose, 10 nM, explaining the equivalent efficacy resulting from the FLT3 inhibition. To better understand how much the efficacy of compound **15** within the in vivo experiments, a xenograft model of MV4-11 cells was constituted to reveal that therapy with compound **15** (given as a single daily oral dose of 20 mg/kg for 21 days) afforded quick and comprehensive lessening of tumors' mass in all tested mice relative to the PBS standard group; additionally, all the cancer bulkiness died out within day 15, founded on the previously detected tumor bulkiness with no weightiness wastage or somewhat systemic poisonousness within the therapy term.

**Fig. 7:**
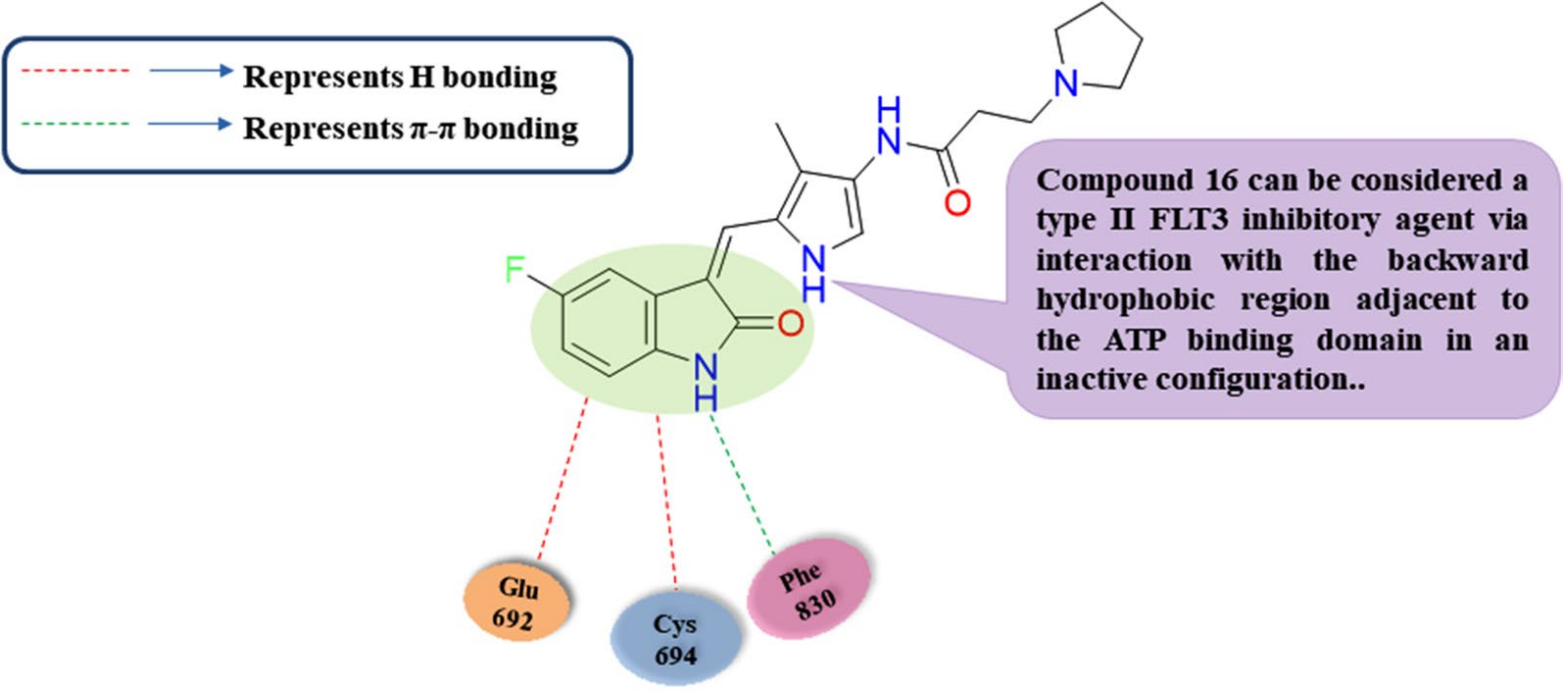
Simple 2D diagram of the predicted binding modes of compound **16** into DFG-out FLT3 kinase homology (PDB: 4XUF); illustrating the two main hydrogen bonding interactions of compound **16** with Glu692 and Cys694, and one π-π stacking interaction with Phe830 in the DFG-out model

Recycling the approved drugs for FLT3 circumvention lessens the cost and time of drug development and allows identifying scaffolds of well-known safety margins, pharmacokinetics, and pharmacodynamic profiles. For example, Wang et al. reported synthesis and investigation of the FLT3 circumvention ability of compound **16** (Sunitinib-based scaffold) upon carrying out a western blotting assay. It was found that compound **16** had an unusual diminishing activity over the innate wild FLT3 with IC_50_ equals to 1.8 nM, the mutated ITD-FLT3 with IC_50_ equals 0.8 nM, and the mutated D835Y-FLT3 with IC_50_ equals to 1.9 nM **(**Table 3**)** [86]. Moreover, compound **16** afforded higher selectivity over c-KIT kinase (over 500 thresholds) with IC_50_ equals 1.8 nM over wild FLT3, and IC_50_ value higher than 1000 nM over c-KIT, additionally over three thresholds selectivity than Sunitinib, three thresholds selectivity than Quizartinib and over twenty thresholds selectivity than Midostaurin, illustrating compound **16**’s higher safety margin. 3D docking study of compound **16** fitting into DFG-out FLT3 kinase homology (PDB ID: 4XUF) elucidates that compound **16** had an efficient co-structure with the binding region of FLT3 DFG-out conformation wherein the oxindole ring not only had two main hydrogen bonding interactions with Glu692 and Cys694 with shorter distances of 1.9 and 2.4 Å, respectively in the hinge domain, also compound **16** had a strong π − π stack interaction with Phe830 in the DFG-out model, and extra π- π interactions in the hydrophobic pocket resulting in ligand binding **(**Fig. 7**)**. Consequently, compound **16** can be considered a type II FLT3 inhibitory agent via interaction with the backward hydrophobic region adjacent to the ATP binding domain in an inactive configuration. Furthermore, to evaluate compound **16** selectivity for FLT3 kinase over c- KIT kinase, a computational study of compound **16** interaction with c- KIT kinase was carried out using a DFG-in configuration (PDB ID: 1T46). It was found that compound **16** was unable to form any hydrogen bonding with the binding domain’s amino acid residues; however, Sunitinib has two hydrogen-bonding interactions with Cys673 in the hinge region, in addition to imatinib as a selective c-KIT hampering agent, had three hydrogen bonding interactions with Glu640, Cys673, and Asp810, respectively. So, it is confirmed that compound **16** had significant circumvention selectivity on FLT3 kinase over c-KIT kinase. Compound **16** showed powerful proliferation circumvention on FLT3-ITD- MV4-16 cells with IC_50_ equal to 23.5 nM and MOLM-13 with IC_50_ equal to 35.5 nM. Furthermore, compound **16** revealed about 67.8 and 60.3% hampering percentage versus Sunitinib, in addition to Quizartinib resistant cell lines, respectively, upon using 1000 nM as compound **16** dosage. Consequently, compound **16** had a fundamental efficacy versus Sunitinib and Quizartinib-resistant acute myeloid leukemia cells. Besides, upon carrying flow cytometry assay to detect the dose-dependent apoptosis induction ability of compound **16**, it was evaluated that 100 nM dosage of compound **16** afforded apoptosis induction percentages of about 25.1 and 54.6% in MV4-16 and MOLM-13 cells, respectively. Compound **16**'s apoptosis effect was more potent than the effect of the same Sunitinib dosage.

**Fig. 8:**
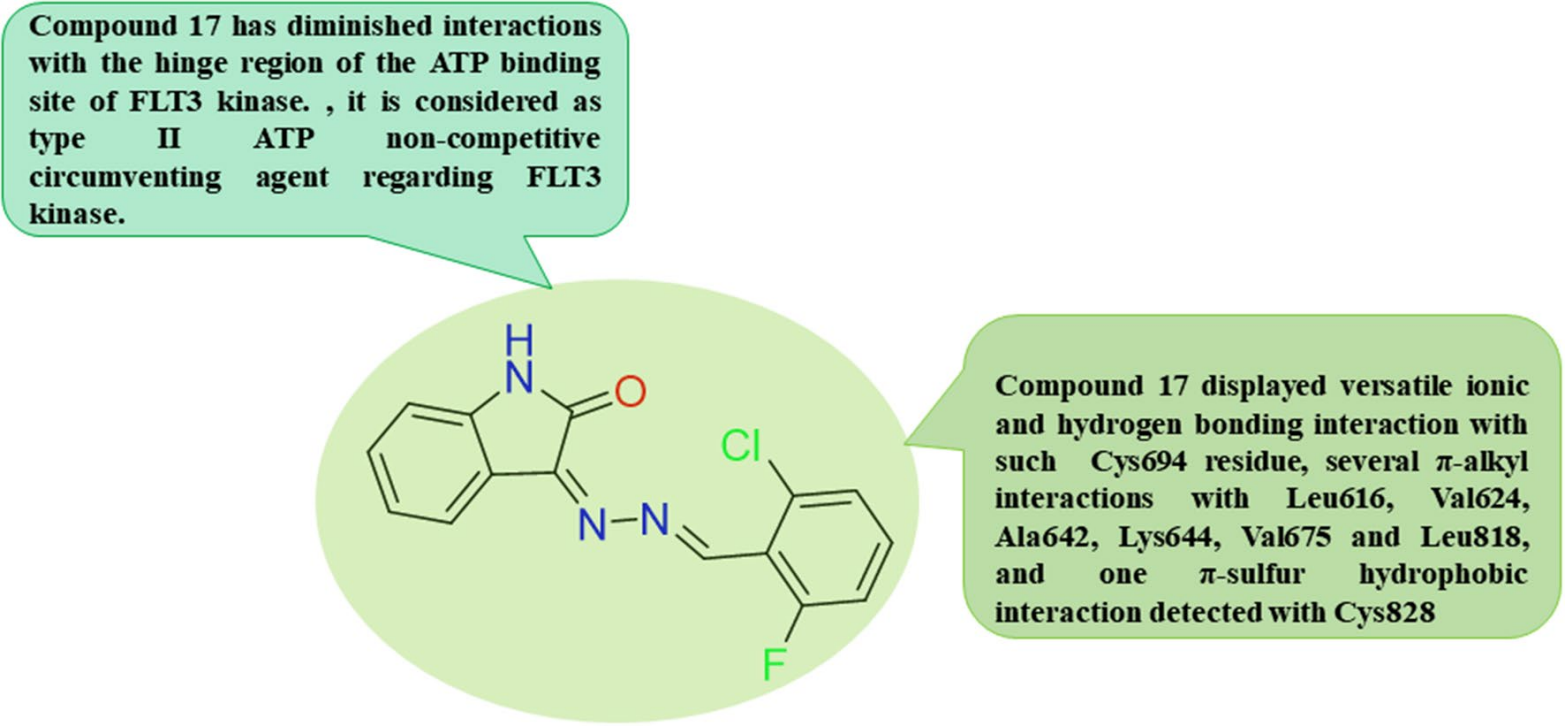
Structure–activity relationship of compound **17**

Among the discovery of novel scaffolds with multiple kinase inhibitory activities versus various kinases, including FLT3 kinase, Al-Salem et al. in 2021 synthesized many oxindole-based derivatives with versatile kinase inhibitory activity, specifically, compound **17** [87]. Compound **17** showed circumvention of different tyrosine kinases, including CDK-2 with IC_50_ equal to 0.301 µM, which was about half the threshold lower than recognized kinase inhibitor imatinib with IC_50_ equal to 0.131 µM and comparable to the primary FLT-3 hampering agent Sunitinib with IC_50_ equal to 0.262, and versus EGFR, VEGFR-2 (KDR), and FLT-3 with IC_50_s equal to 0.369, 0.266, and 0.546 µM, respectively **(**Table 3**)**. Molecular modeling studies were carried out for compound **17** versus EGFR (PDB ID: 6DUK), VEGFR-2 (PDB ID: 3VHE), and FLT-3 kinases (PDB ID: 6JQR). Regarding fitting compound **17** into the EGFR kinase domain, compound **17** affords one hydrogen interaction with Phe856, multiple hydrogen bonds with Asp855 and Phe856 presented in the ATP binding domain and DFG model, and various hydrophobic interactions with many amino acids' residues in the ATP binding region. Furthermore, fitting compound **17** with the VEGFR-2 kinase region elucidates forming two hydrogen bonds with the gatekeeper Cys919 residue, π-π T molded interaction with DFG motif Phe1047 residue: and numerous π-alkyl hydrophobic interactions with many amino acids’ residues in the ATP binding region. Besides, compound **17** displayed successful fitting into the FLT-3 kinase domain, including versatile ionic and hydrogen bonding interaction with such Cys694 residue, several π-alkyl interactions with Leu617, Val624, Ala642, Lys644, Val675, and Leu818, and one π-sulfur hydrophobic interaction detected with Cys828 **(**Fig. 8**)**. In relation, the co-structure of FLT-3 with Quizartinib exhibited that interactions with the DFG motif Phe830 residue and the hinge region Phe691 residue are fundamental for like this kinase inhibition. Compound **17** had diminished interactions with the hinge region of the ATP binding site of FLT3 kinase. Herein, compound **17** represented a type I ATP competitive hampering agent versus EGFR and VEGFR-2 kinases; however, it was considered a type II ATP non-competitive circumventing agent regarding FLT3 kinase. Moreover, it was detected higher potential of compound **17** versus human breast cancer (MCF7) with an IC_50_ value of 3.56 µM relative to the standard anticancer medicine, doxorubicin, with an IC_50_ value of 3.10 ± 0.29 µM.

**Fig. 9:**
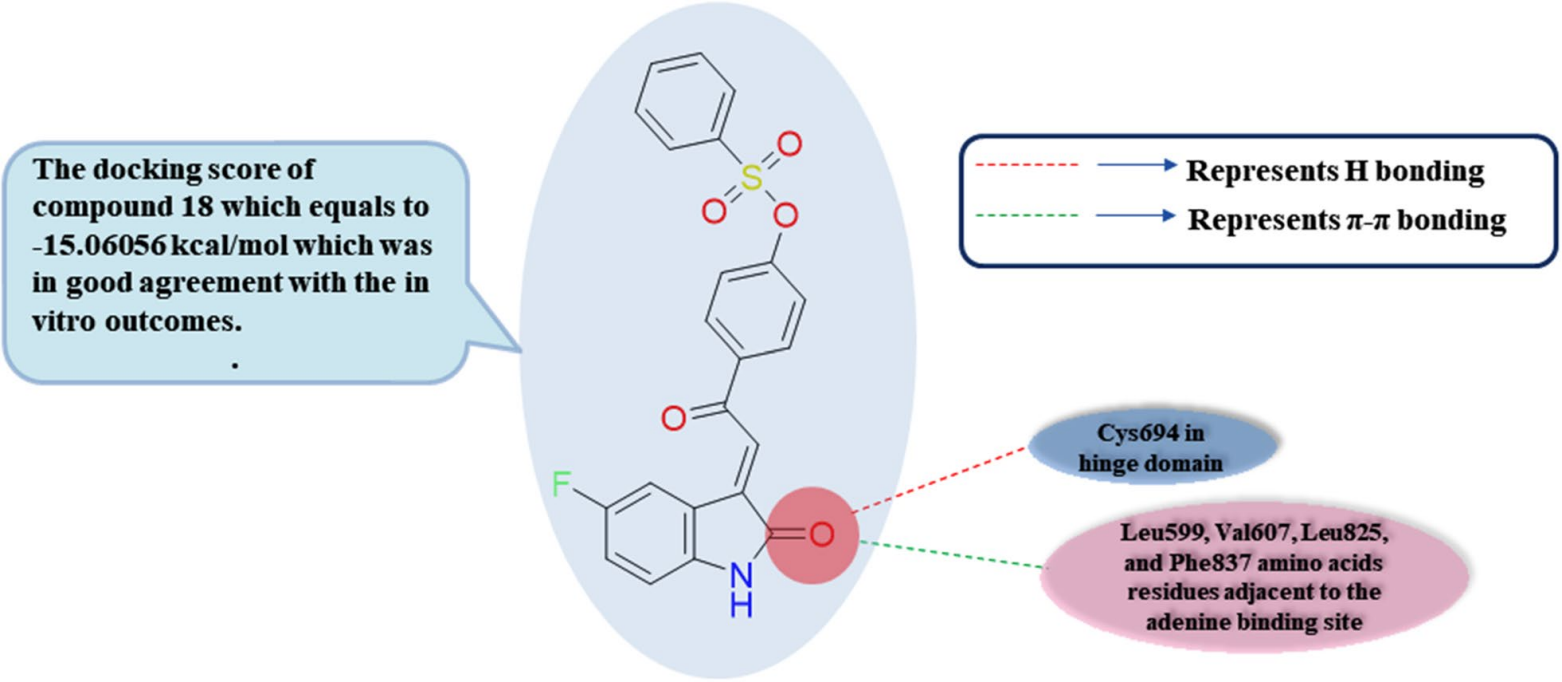
Simple 2D diagram of the predicted binding modes of compound **18** the ATP binding domain of PDGFRα (PDB: 5GRN); illustrating the two main hydrogen bonding interactions of compound 18 with Cys694 in hinge domain, and various π-π stacking interactions with Leu599, Val607, Leu825, and Phe837 amino acids residues adjacent to the adenine binding site

Amongst continuous research studies work to combat several cancer cells' resistance via circumvention of diverse kinases, including FLT3, PDGFRα, VEGFR-2 (KDR), and Aurora A and B; in 2021, El-Hussieny et al. stated compound **18** that afforded versatile tyrosine kinases' inhibitory activity including PDGFRα, and FLT3 that are fundamental for remedy of various leukemia kinds with IC_50_ equal to 24.15 nM meaningfully less than Sunitinib IC_50_ value equal to 58.93 nM, and IC_50_ equal to 74.85 nM less than Sunitinib IC_50_ value of 38.8 nM, respectively **(**Table 3**)** [88]. Besides, dual circumvention of VEGFR2 and Aurora A/B is a promising approach for improving antiproliferative activity versus multiple cancer species; compound **18** showed hampering activity versus VEGFR2, Aurora A, and B with IC_50_ value of 46.65 nM relative to Sunitinib IC_50_ value of 42.33 nM, IC_50_ equal to 16.83 nM higher than Sunitinib IC_50_ value of 48.42 nM, and IC_50_ equal to 29.38 nM less than Sunitinib IC_50_ value of 17.33 nM, respectively. Docking studies were carried out for compound **18** upon its fitting into the ATP binding domain of PDGFRα (PDB ID: 5GRN**)**; it was detected that the carbonyl group of the 2-oxindole ring of compound **18** forms a critical hydrogen bonding interaction with the group of the NH moiety of Cys677 amino acid residue in hinge domain, besides π- π interactions with the various amino acids residues adjacent to the adenine binding site involving Leu599, Val607, Leu825, and Phe837 **(**Fig. 9**)**. These docking results were imitated on the docking score of compound **18** which was -15.06056 kcal/mol that were in good agreement with the in vitro outcomes. Furthermore, compound **18** displayed remarkable antiproliferative activity versus CCRF-CEM, and SR leukemia cell lines with IC_50_ values equal to 6.84 and 2.97 µM, respectively, relative to Sunitinib with IC_50_ values equal to 5.37 and 10.36 µM, respectively. The anticancer activity of compound **18** versus five leukemia cancer cell lines, specifically CCRF-CEM, HL-60 (TB), K-562, MOLT-4, and SR, was evaluated with growth inhibition percentage values equal to 91.32, 66.93, 50.89, 59.32, and 90.58, respectively. Regarding cell cycle arrest analysis in SR leukemia cells, it was found that compound **18** causes cell cycle arrest at the S and pre-G1 phases with a rise by 1.87 and 20.45 thresholds, respectively, and concurrent lessening % at G0-G1 and G2/M cycles by around 0.84 and 0.54 thresholds. Furthermore, concerning cell cycle arrest in CCRF-CEM leukemia cells, compound **18** caused a valuable reduction in cell proportion at G2/M and pre-G1 cycles with a rise of 2.78 and 17.62 thresholds, respectively, in addition to rapid decline at G0-G1 and S phases by nearly 0.75 and 0.69 thresholds, respectively relative to untreated cells. Also, compound **18** exhibited a significant elevation in apoptotic cells ratio in CCRF-CEM and SR leukemia cells from 1.71 to 28.42% and from 0.19 to 25.93%, respectively, compared to the untreated cells. Compound **18** increased necrotic cells’ proportions in CCRF-CEM and SR leukemia cells with about 9.76- and 6-thresholds rise, respectively, relative to the control untreated cells.

Interestingly, Bender et al., in 2022, reported synthesis and investigation of the FLT3 circumvention ability of compound **19** that showed powerful proliferation circumvention on FLT3-ITD- MV4-16 cells with IC_50_ of 4.3 μM and THP‐1 cells with IC_50_ of 8.7 μM at 72 h, with growth inhibition % of 91, and 39, respectively [89]. Thus, it showed two-threshold selective inhibition against the oncogenic ITD mutation. High-throughput protein profiling elucidated the decreased levels of the growth factors IGFBP-2 and‐4 with the obstruction of various apoptotic inhibitors such as Survivin. p21 with cellular stress signaling pathways was characterized by intensified expression of HSP proteins and TNF-β. Mechanistically, in vitro FLT3 inhibition assay revealed that compound **19** can inhibit FLT3 kinase with IC_50_ values of 2.49 μM. 3D docking study of compound **19,** fitting into the co-crystalized structure of FLT3 and Gilteritinib (PDB ID: 6JQR), elucidates that compound **19** could fit into the FLT3-ATP binding site with the formation of highly stable complexes as confirmed by molecular dynamics simulations study carried out later. The key interactions observed in the crystal complex FLT3 appeared in the docking pose of cis-**19** with two essential hydrogen bonds, respectively, with Glu692 and Cys694, which adopt the amide moiety of the β-lactam. The two aromatic moieties are fitted into the enzymatic pocked similarly to the crystallographic ligand. Indeed, the oxindole ring can potentially occupy the enzymatic pocket occupied by the piranyl-aminopyrazine carboxamide core center of the crystallographic ligand.

To elucidate the efficacy of the oxindole-based scaffolds (**11**, **12**, and **14–18**), many aspects have been investigated, such as potency and selective inhibition towards wild and mutated FLT3 types rather than other kinases, cytotoxicity versus FLT3 expressing leukemic cell lines as Molm-13 and MV4-11, cell cycle arrest ability, and anti-apoptotic capability. Also, xenograft models had been constructed for optimum in vivo illustration for the antiproliferative, physicochemical, and tolerability properties of those scaffolds upon FLT3 harboring AML patients like compounds **12** and **15**. Compound **13** was the only one investigated for its physicochemical properties, such as its lipophilicity (logP) which could be more helpful for better oral bioavailability and biological activity of this compound. Therefore, all previously mentioned oxindole-based compounds should be fully investigated through the in vivo xenograft studies and accurate detection of pharmacokinetic and pharmacodynamic properties to facilitate the entrance of those scaffolds into clinical trials as promising FLT3 inhibitors versus FLT3 AML cells.

## Indazole based FLT3 inhibitors

Several reports show indazoles have shown anticancer activities [90, 91]. Indazoles, as a core structure of the multi-kinase inhibitor Linifanib (**20**), were also used to design several kinase inhibitors, including FLT3, where the ring could fit into the hinge region of the FLT3 kinase enzyme [92]. Briefly, Linifanib (**20**) is an orally active indazole-based multi-target RTK inhibitor including KDR, CSF-1R, and PDGFR-β [93] (Table 4**)**. Surprisingly, Linifanib displays a type II mode of binding with FLT3 receptor [13]. Phase II clinical studies evaluated that Linifanib can be efficiently utilized in therapy for cases with advanced or refractory colorectal carcinoma with a highly expressed rate of k-Ras mutations [93]. Multiple research studies have recently emphasized diaryl ureas as efficient antiproliferative agents; for instance, Linifanib as a diaryl ureas-based scaffold achieved great attention as a promising antiproliferative agent. Mechanistically, Linifanib can hamper multiple kinases' activity, such as FLT3, VEGFR-2, and PDGFR-β, with its IC_50_ values of 4, 4, and 66 nM, respectively [94]. In 2016, the higher margin of safety, tolerability, and pharmacokinetic and pharmacodynamic (PK/PD) properties of Linifanib were evaluated in phase I trial individually (group A) or combined with Cytarabine (Ara-C) (group B) in forty-five patients collected from five different sites in the United States (divided into 29 in group A and 18 in group B) with relapsed/refractory AML [95]. Linifanib showed a good safety profile and was tolerated well [95]. Side effects were as well known for refractory/relapsed AML and were identical to those previously mentioned in other Linifanib trials as adverse drug effects following VEGFR inhibition, namely fatigue, diarrhea, skin toxicities, proteinuria, and hypertension [96–98]. However, myelosuppression has been noted with other RTK inhibitors; it was not reported here [99], potentially due to the kinase specificity of this drug [96]. Toxicities in group B were largely related to Linifanib and did not differ from group A [95]. The pharmacokinetic (PK) levels of Linifanib were dose-proportional from 10 to 20 mg in both groups [98]. Based on the safety profile and PK data, the recommended phase 2 dose (RPTD) of Linifanib as a single agent was 15 mg, and in combination with Ara-C, it was 10 mg in investigated patients with relapsed/ refractory AML [95]. In vitro, Linifanib was a potent FLT3 inhibitor with an IC_50_ value of 100 nM in normal human blood-spiked AML patients [100] and potentiated FLT3 kinase inhibition compared with other agents like Sorafenib [96]. Consequently, future research studies are required to elucidate these clinical signals in FLT3 wild-type AML patients. Moreover, the favorable higher margin of safety and the potent activity of Linifanib against FLT3 -ITD AML; clarify the higher necessity of further in vivo and clinical future studies of this agent.

**Table 4:** Indazole-based FLT3 inhibitors

Compound name and its structure	Description		Refs.
	Target	FLT-3, VEGFR2 (KDR), and PDGFR-β	[92–95]
	Binding mode to FLT3 kinase	Type II	
	FLT3 Potency (IC_50_)	4, 4, and 66 nM, respectively	
	In vitro targeted AML cells (IC_50_)	0.69 μM (MV4-16)	
	Target	c- Kit, PDGFR-ẞ, and FLT3	[101]
	Clinical development	In vivo xenograft Studied	
	FLT3 Potency (IC_50_)	68.5, 150, and 375 nM, respectively	
	In vitro targeted AML cells (IC_50_)	1.17, 1.34, and 1.77 μM (SK-MEL-3, NB-4, and SK-MEL-31 cancer cells, respectively)	
	Target	FLT3	[102]
	Binding mode to FLT3 kinase	Type I	
	FLT3 Potency (IC_50_)	9 nM	
	In vitro targeted AML cells (IC_50_)	174 nM (FLT3-ITD- MV4-16)	
	Target	FLT3, D835H-FLT3, D835Y-FLT3, ITD-FLT3, K663Q-FLT3, N841I-FLT3, KIT, and PDGFR	[103]
	Binding mode to FLT3 kinase	Type II	
	Residual control percentage (%ctrl) versus protein kinases	0.15, 0.05, 0.1, and 0.05% (FLT3, D835H-FLT3, ITD-FLT3, and KIT, respectively)	
	In vitro targeted AML cells (IC_50_)	5, 16, and 198 nM (FLT3-MOLM18, PDGFRa-T674M-Ba/F3, and Kit-T670I-Ba/F3 cells, respectively)	
	Target	(DYRK1-a, AMPK-a1, and RSK-1 (moderator for the anti-apoptotic purpose of FLT3-ITD in AML cells)	[104]
	Residual control percentage (%ctrl) versus protein kinases	4, 4, and 8%, respectively	
	In vitro targeted AML cells (IC_50_)	From 2 to 3 μM/L (Molm-18, heterozygous FLT3-ITD, and homozygous FLT3-ITD-MV4-16 cells)	

As a result of the quick emergence of versatile resistant tumor cell lines versus many reported kinase inhibitor drugs, it was obligatory to invent multiple kinase inhibitor scaffolds. In relation, Lu et al. in 2015 discussed that compound **21** at a dosage of 10 μM could act as multiple kinases circumventing agent versus c- Kit, PDGFR-ẞ, and FLT3 with binding affinity (K_d_) values of 68.5, 150, and 375 nM, respectively **(**Table 4**)** [101]. Consequently, regarding the control percentage (%Ctrl) representing the lysed kinases indicating lower percentage values associated with efficient binding with compound **21** against c-Kit, PDGFRẞ, and FLT3 with percentage values of 0, 0.05, 2.8%, respectively, therefore compound **21** could be utilized to combat different cancer kinds by lessening as possible resistance versus them. Furthermore, the antiproliferative efficacy of compound **21** was tested upon incubation for 72 h versus 17 cancer cell types, and cell viability was evaluated by CCK8 analysis. It was clear that compound **21** afforded a robust antiproliferative activity with IC_50_ values oscillating between 1.17 to 6.84 μM in these cell lines, specifically SK-MEL-3, NB-4, and SK-MEL-31 with IC_50_ equal to 1.17, 1.34, and 1.77 μM, respectively **(**Table 4**)**. This fundamental potent antineoplastic activity of compound **21** was attributed to the circumvention of c-Kit, PDGFRẞ, and FLT3 tyrosine kinases and their down proceeding signaling cascades in tumor cells. The in vivo experiment was constructed using kunming mice engrafted with S180 cancer cells to demonstrate that using 25 mg/kg as an oral dosage of compound **21** for 17 days, the cancer mass engrafted with S180 cells was ultimately minimized by about 67.3% in addition to the cancer bulkiness. Interestingly, oral ingestion of compound **21** was highly safe for mice with no observable body mass loss, which elucidates that oral consumption of compound **21** in mice successfully prohibited the progress of engrafted S1cells without any apparent general toxicity.

**Fig. 10:**
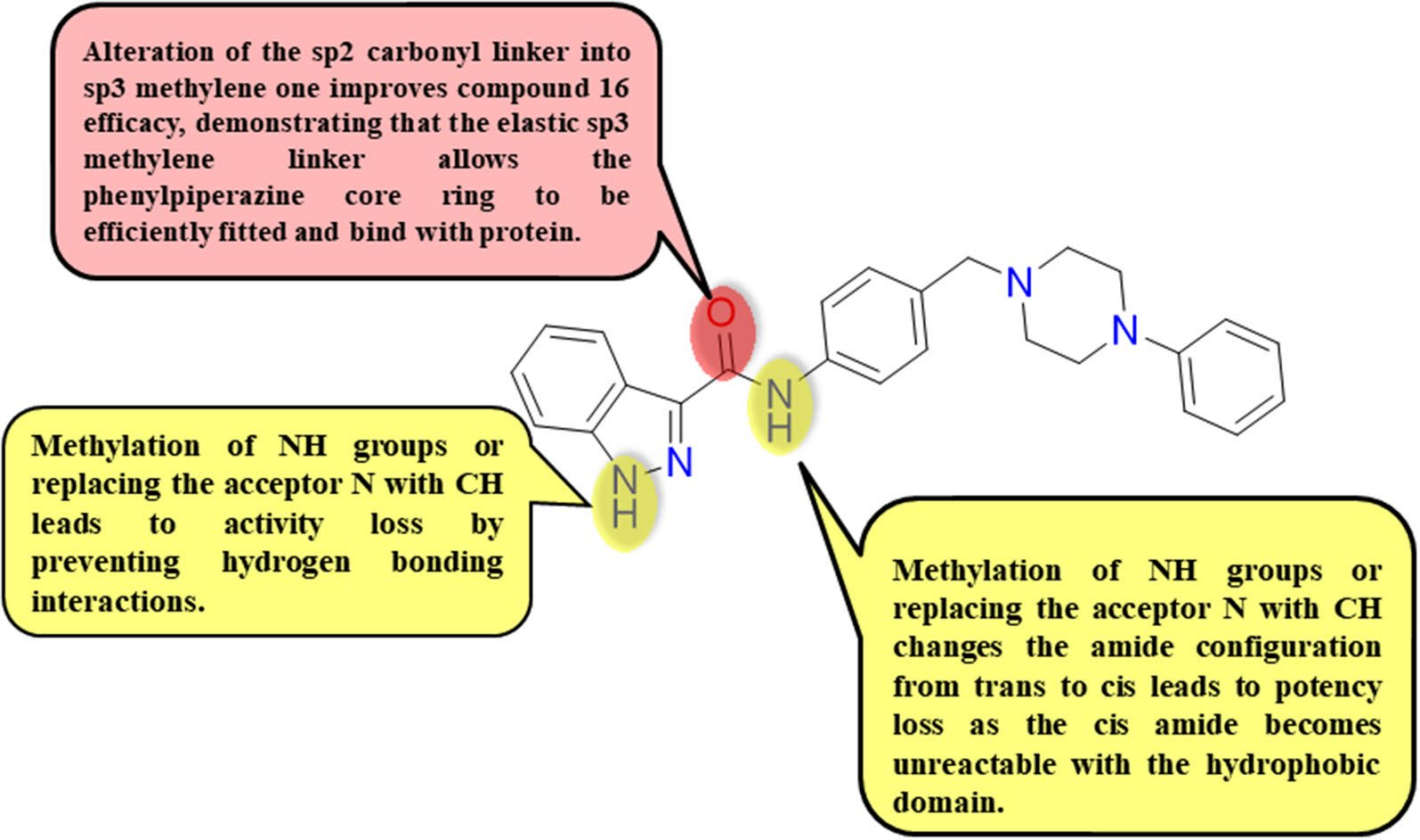
Structure–activity relationship of compound **22** for fitting with the DFG-in hinge region of CDK2/1 analog co-structure model (PDB: 2VTI)

In 2017, Nakano et al. reported compound **22**, which can be expressed as a highly potent and selective FLT3 inhibitor with IC_50_ equal to 9 nM [102]. The impact of higher selectivity of compound **22** on FLT3 kinase rather than about 49 other kinases appeared upon testing compound **22** at a dosage of 0.450 µM (fifty folds greater than the IC_50_ value of FLT3), showing lessening of activity inhibition percentages of both TRKA and HGK from 81 to 63%. In contrast, the IC_50_ of TRKA equal to 187 nM (fifteen times selectivity upon FLT3) and of HGK equal to 410 nM (forty times selectivity upon FLT3); besides, the other 47 tested kinases were hampered by lower than 40% **(**Table 4**)**. 3D computational docking studies of compound **22** elucidate that binding of compound **22** to the DFG-in hinge region of FLT3, as in the beforehand reported CDK2/1 analog co-structure model (PDB ID: 2VTI), owing to the reported fact of FLT3-1 was alike in its identity and interactions to the CDK2/1 analog. In relation, at least three hydrogen bonds in the hinge region are necessary for inhibitory activity, so methylation of NH groups or replacing the acceptor N with CH leads to activity loss. Moreover, the methylation of NH groups would lead to activity loss by preventing hydrogen bonding interactions, and changing the amide configuration from trans to cis leads to potency loss as the cis amide was unable to fit into the hydrophobic domain **(**Fig. 10**)**. Alteration of the sp2 carbonyl linker into sp3 methylene one ameliorated compound **22** efficacy, demonstrating that the elastic sp3 methylene linker permits the phenylpiperazine core ring to be efficiently fitted and bind with protein. To further investigate compound **22** potency, it was tested on its inhibition efficacy on the progress of FLT3-ITD- MV4-16 cells exhibiting IC_50_ equal to 174 nM, while insignificant circumvention on the development of WI-38 (normal human diploid lung fibroblast cell), as a testing technique for systemic toxicity. Moreover, the autophosphorylation circumvention ability of FLT3 was examined upon 1.5 h incubation of compound **22** in MV4-16 cell lines, then lysed and carrying out western blotting assay, relying on the reported fact that FLT3 can phosphorylate Tyr-596; it was found that compound **22** in a dose-dependent manner can hamper FLT3 phosphorylation. Apoptosis induction of compound **22** was tested by annexin V staining**;** it was detected that compound **22** upon 48 h incubation encourages apoptosis in MV4-16 cell lines at 0.16 μM concentration nearly in the same manner such as Quizartinib as a standard FLT3 inhibitor; so, compound **22** represents a favorable scaffold requiring further optimization and development as an efficient FLT3 inhibitor.

Among many trials to get highly potent and multi-targeted kinases inhibitor to combat highly aggressive and resistant tumor types, Deng et al. reported the amino indazole derivative compound **23** and scanned his circumvention activity at a dosage of 10 μM versus a series of 402 kinases via following the in vitro ATP- competition binding technique, it was demonstrated that compound **23** was strongly bonded with many kinases, especially FLT3, D835H-FLT3, D835Y-FLT3, ITD-FLT3, K663Q-FLT3, N841I-FLT3, KIT, and PDGFR, with percent control proportions (% ctrl lower values indicate strong inhibitory capability) of 0.15, 0.05, 0.0, 0.1, 0.0, 0.0, 0.05, and 0.0%, respectively **(**Table 4**)** [103]. Moreover, various kinases strongly bonded with compound **23**, such as FLT3, KIT, and PDGFR, are commonly targeted by type II DFG-out scaffolds. Also, it was found that compound **23** displayed more than one thousand thresholds selectivity towards FLT3, and Kit- T670I expressing cells revealed the fact that compound **23** was a selective cytotoxic agent, especially towards FLT3 expressing cells. Furthermore, compound **23** displayed higher antiproliferative activity against FLT3-MOLM18, PDGFRa-T674M-Ba/F3, and Kit-T670I-Ba/F3 cells with EC_50_s equal to 5, 16, and 198 nM, respectively. Consequently, compound **23** can be described as a highly potent and selective inhibitor versus FLT3 and PDGFR kinases.

Bjørnstad et al. in 2019 reported compound **24**, which was tested at a dosage of 1 mmol/L versus 50 protein kinases, illustrating that this compound afforded highly observable activity against both Pim1&3, additionally at 1 μM concentration of compound **24,** noticeable inhibition versus DYRK1-a (phosphorylation regulated kinase 1A) with the remaining activity of 4%, AMPK-a1 (AMP-activated kinase which is a stress feeler in cells, also incorporated in the autophagy initiation, and can act as a metabolic stress protector for AML-cells) with the remaining activity of 4%, and RSK-1 (ribosomal protein S6 kinase A1 which is a moderator for the anti-apoptotic purpose of FLT3-ITD in AML cells) with the remaining activity of 8% **(**Table 4**)** [104]. The higher efficacy and tendency of compound **24** in AML cells may be attributed to the circumvention of anti-apoptotic mediators, specifically RSK1 and AMPK, with its concurrent pan-Pim kinase inhibitory activity and this also relies on the fact that AZD1708, which is one of the critical selective Pim kinase inhibitors, was less potent than compound **24** versus tested AML cell lines. Moreover, compound **24** can be considered a highly effective triggering agent for acute myeloid leukemia cells’ death. It can combat versus mutant expressing factors in acute myeloid leukemia cells, namely FLT3-ITD (like in MV4-16 and Molm-18 cells), p53 silencing (like in shp53 Molm-18, and shp53 MV4-16), and overexpressing of Bcl2 protein (like in IPC-81-Bcl2). Cell apoptosis induction of compound **24** was evaluated through the microscopic valuation of the morphology of the nucleus and the metabolic alteration of the WST-1 reagent, which its signal decay was associated with apoptosis induction. It was found that the cytotoxicity and cell death induced by compound **24** appeared as distinctive apoptotic morphology features, for example, cell shrinkage, nuclear condensation, and fragmentation with EC_50_ = 1.23 μMol/L; in addition to potent inhibition of both the FLT3-ITD anti-apoptotic factor RSK1 and AMPK in AML cells affording more circumvention of leukemia cells’ propagation. Eventually, upon following the autophosphorylation hampering activity of compound **24**, it was evaluated that compound **24** lessened Pim kinases’ phosphorylation; in relation, PIM kinases are well known to be one of the down proceeding signaling cascades resulting from FLT3 phosphorylation. Consequently, compound **24** exhibited much more potency versus the FLT3-ITD mutated cell lines such as Molm-18, heterozygous FLT3-ITD, and homozygous FLT3-ITD-MV4-16 cells with EC_50_ fluctuating between 2 and 3 μMol/L.

Regarding indazole-based scaffolds (compounds from **21–24**), they acted as multiple kinases inhibitors, including FLT3 (compound **21**) or inhibitors for other kinases (Pim) that are one of down proceeding signaling cascades from FLT3 phosphorylation (compound **24**); that could result in many off-target side effects. Also, antiproliferative activity and apoptosis induction properties were examined specifically for compounds **22** and **24**. In vivo studies were carried out for compound **21** that elucidated its potent cytotoxicity versus engrafted S1 cancer cells rather than normal cells and its higher tolerability with no significant toxicity signs and higher overall survival rate. Therefore, all those indazole-based scaffolds had to be fully tested for their physicochemical properties, tolerability, cell cycle arrest, and apoptosis induction capabilities to allow affording more potent and selective FLT3 inhibitors against different FLT3 overexpressing AML cells with higher. Besides, several resistance mechanisms versus indazole-based compounds such as FLT3-ITD, anti-apoptotic mediators (AMPK and RSK1), and p53 silencing should be well screened to afford more efficient FLT3 inhibitors.

## Benzimidazole based FLT3 inhibitors

Over time, benzimidazole and its derivatives represent essential core scaffolds in various biologically active heterocyclic compounds with various pharmacological activities, like anticancer agents [105, 106]. For instance, Crenolanib (**25**) is a benzimidazole-based small structured TKI initially established as a PDGFR inhibitor (Table 5**)**. Through the in vitro assays, including different cell lines and the in vivo xenografted mouse models, Crenolanib exhibited powerful inhibitory activity versus both FLT3-ITD and FLT3-TKD. This character is auspicious given some FLT3-TKD mutated forms, including D835, which combat the circumvention abilities of most well-known FLT3 inhibitors [107]. Mechanistically, Crenolanib shows a type I binding mode with the FLT3 receptor [13]. In an experimental study of Crenolanib as a PDGFR restrainer, its margin of safety, stability, and pharmacokinetic properties have been briefly evaluated to facilitate moving forward toward clinical studies of Crenolanib as an FLT3 hampering agent in AML cases [108]. Levis et al. have estimated Crenolanib's concentration in the plasma of the cases enrolled in these clinical trials, which was enough to hamper both FLT3-ITD and mutated FLT3-D835 [108]. About 41% of patients in phase II clinical trials responded well after a single crenolanib treatment [109]. Due to the outstanding preclinical outcomes, Crenolanib entered clinical trials to elucidate its safety and tolerability with standard traditional chemotherapy in patients with mutated FLT3 AML cells (NCT02283177) [110]. Of twenty-two patients, nineteen had an FLT3-ITD mutation, while the remaining three had an FLT3-D835 mutation, and 88% of patients acquired a complete alleviation in this study. Crenolanib has already entered into phase III clinical trials to examine its efficacy in combinatorial chemotherapy with chemotherapy compared to using chemotherapy individually in investigated cases with relapsed mutated AML [110]. Another phase III trial has been done testing Crenolanib versus Midostaurin following induction and consolidation chemotherapy in patients with newly diagnosed FLT3 harboring AML cell lines [68].

**Table 5:** Benzimidazole-based FLT3 inhibitors

Compound name and its structure	Description		Refs.
	Target	FLT3, and PDGFR α/ẞ	[80, 81, 108, 109]
	Binding mode to FLT3 kinase	Type I	
	Mutation point	F691L	
	FLT3 Potency (IC_50_)	2, 1.3, 2 nM (FLT3-ITD (in MV4-16and MOLM 15), FLT3-ITD (in Ba/F3 cells), FLT3-wt (in SEMK2 cells))	
	In vitro targeted AML cells (IC_50_)	1.3 and 4.9 nM (in MV4-16, and MOLM 18, respectively)	
	Target	FLT3	[111]
	Binding mode to FLT3 kinase	Type I	
	FLT3 Potency (IC_50_)	495 nM	
	Target	FLT3-wt, FLT3-ITD, and FLT-TKD-D835Y	[112]
	Binding mode to FLT3 kinase	Type I	
	FLT3 Potency (IC_50_)	43.8, 97.2, and 92.5 nM, respectively	
	In vitro targeted AML cells (IC_50_)	38.8 and 54.9 nM (in MV4-16 (expressing FLT3-ITD), and MOLM-18(expressing FLT3-ITD), respectively)	

**Fig. 11:**
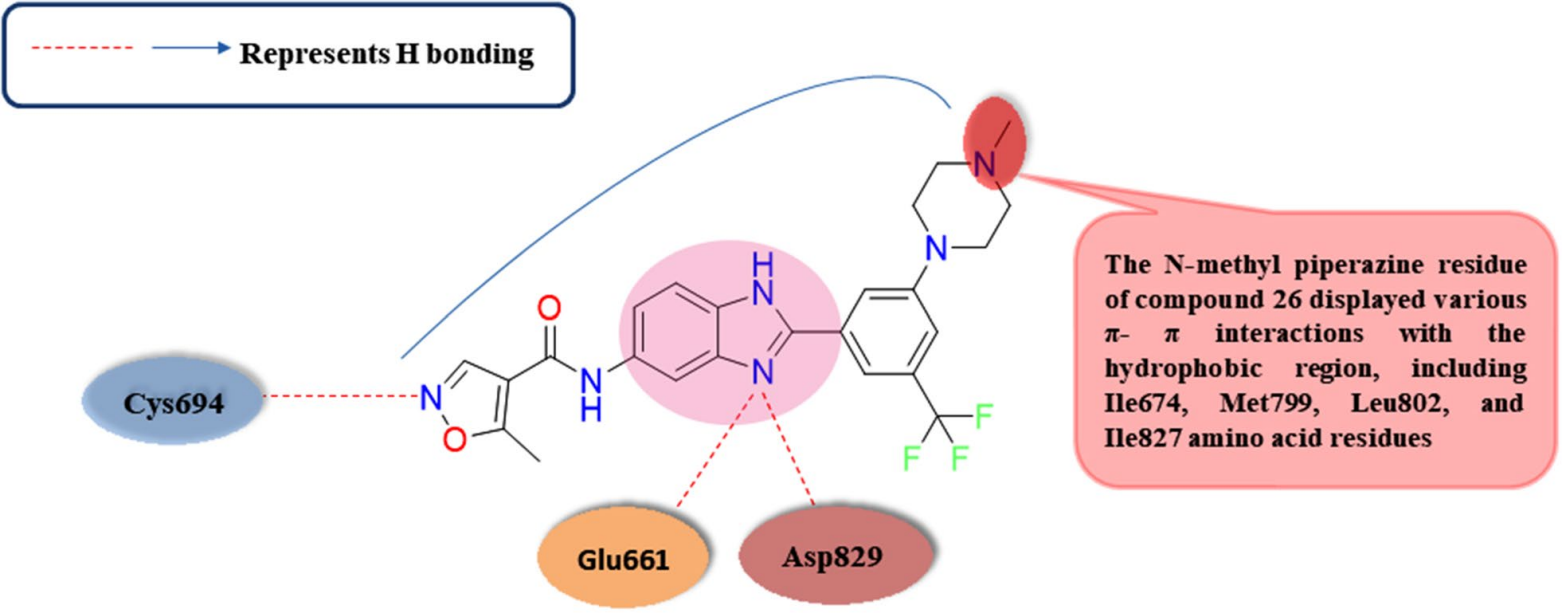
Simple 2D diagram of the predicted binding modes of compound **26** into the ATP-binding site of FLT3 (PDB: 4RT7)

Im et al. in 2019 studied the inhibitory activity of benzo-imidazole derivative **26** at a dosage of 10 μM versus protein kinases tested versus a series of more than 35 several kinases. Specifically, it was detected that compound **26** exhibited outstanding selective hampering activity percentages against FLT3 and FLT3- ITD of 89.97, and 88.48%, respectively, without any observable activity versus other protein kinases **(**Table 5**)** [111]. Moreover, a western blotting assay for the detection of the ability of compound **26** to circumvent phosphorylation of protein kinases, especially FLT3, was carried out and it revealed that compound **26** afforded highly critical hampering activity versus FLT3 with IC_50_ equal to 495 nM. Computational docking studies demonstrate that compound **26** can selectively fit into the ATP-binding site of FLT3 (PDB ID: 4RT7) through 3 main hydrogen bonding, π- π, a π- cationic, and ionic interactions. Briefly, hydrogen bonds represented one hydrogen bond between the 5-methylisoxazole nitrogen as a linker with the NH group of Cys694 and two hydrogen bonds between the benzimidazole ring of compound **26** and the oxygen of Glu661 and another with the NH group of the backbone Asp829 **(**Fig. 11**)**. Additionally, the N-methyl piperazine residue of compound **26** displayed various π- π interactions with the hydrophobic region, including Ile674, Met799, Leu802, and Ile827 amino acid residues.

M. E. Dokla et al. in 2022 reported compound **27** as a selective inhibitor versus wild FLT3, FLT3-ITD, and FLT-TKD-D835Y mutated protein kinases with IC_50_s equal to 43.8, 97.2, and 92.5 nM, respectively **(**Table 5**)** [112]. Compound **27** can act as an efficient protein kinase inhibitory agent against both mutated FLT3-ITD and FLT3-D835Y forms in contrast to Quizartinib, which has an insignificant effect, versus mutated forms of FLT3-TKD. Therefore, compound **27** could be considered a type I kinase inhibitor owing to its critical circumventing activity over mutated FLT3-TKD, which stands among the main causes of competence to type II kinase inhibitory activity. Besides, the in vitro selectivity of compound **27** on FLT3 rather than other protein kinases was tested versus a series of 16 protein kinases, especially the off-target tyrosine kinases that efficiently circumvented FLT3 inhibitors such as Quizartinib and Sorafenib; it was demonstrated that compound **27** at a dosage of 1 µM exhibited undetectable inhibitory activity versus FLT3 related kinases involving FLT1, FLT4, PDGFRα, and PDGFRβ. Additionally, it displayed higher than 20 times less efficiency versus c-Kit kinase, whose inhibition leads to strong harmful toxicity, which afforded the serious side effect of myelosuppression. Computational studies for compound **27** on FLT3 kinase indicate that compound **27** can fit into the DFG-in configuration** (**PDB ID: 6JQR**),** forming a hydrogen bonding interaction between benzimidazole moiety and the hinge region Cys694 amino acid residue; however, the acetamido group stretched somewhat into the allosteric site forming one hydrogen bonding with Asp829 amino acid residue with the obvious departure of the allosteric domain vacant, unlike Quizartinib as a type II kinase inhibitor that interacts with the allosteric domain of the DFG-out configuration of FLT3 enzyme (PDB ID: 4XUF). Consequently, compound **27** could be considered a type I kinase inhibitory agent that can interact with the ATP active site and preserve the vital interactions obligatory for inhibitory activity. Moreover, compound **27** affords powerful antineoplastic activity versus FLT3-ITD acute myeloid leukemia cells, including MV4-16, and MOLM-18 with IC_50_s equal to 38.8 and 54.9 nM, respectively **(**Table 5**)**, whereas no fundamental potency versus acute myeloid leukemia cells deficient of FLT3-ITD. Compound **27** affords insignificant cytotoxicity on BNL- normal murine hepatocytes and H9C2- rat cardiomyocytes at concentrations beyond 20 times higher than its IC_50_ value versus FLT3-ITD acute myeloid cell lines demonstrating a higher margin of safety of compound **27**. To evaluate the potency of compound **27** to hamper the down-proceeding signaling cascades such as ERK1/2 and mTOR derived from FLT3-ITD in MV4-16 and MOLM-18 cell lines, a western blot assay was performed for compound **27** using various dosages of 0, 10, 50, and 100 nM for 24 and 48 h of as a therapy incubation period. Eventually, it was found that compound **27** can suppress in dose-relevant mode phosphorylation of both ERK1/2 and mTOR in FLT3-ITD acute myeloid leukemia cells. A flow cytometry assay can evaluate which cell cycle arrest stage that compound **27** with various concentrations (0, 10, 50, and 100 nM) can affect the FLT3-ITD MV4-16 and MOLM-18 cell lines**.** Notably, it is reported that most FLT3 inhibitors can trigger G0/G1 cell cycle arrest; thus, compound **27** can be considered as a selective FLT3 inhibitor by predominantly stimulation of cell cycle arrest at the G0/G1 stage in both MV4-16 and MOLM-18 cells. Moreover, necrosis induction can be confirmed via propidium iodide (PI), a fluorescent DNA-bound dye that can only pass through the cell membranes of both apoptotic and necrotic cells. Herein, flow cytometry assay manifested that compound **27** can stimulate both apoptosis and necrosis of FLT3-ITD acute myeloid cells detected by higher presence percent of PI in MV4-16 and MOLM-18 cells. Ultimately, it is concluded that compound **27** prompted G0/G1 cell cycle arrest, apoptosis, and necrosis of FLT3-ITD expressing acute myeloid cells.

Regarding benzimidazole-based scaffolds (compounds **26** and **27**), they showed higher specificity and selectivity towards both wild and mutated FLT3 types rather than other tyrosine kinases with potent inhibition of both the phosphorylation of FLT3 kinase and the down proceeding signaling cascades derived from FLT3(such as ERK1/2 and mTOR). Therefore, they acted as type I FLT3 inhibitors that could combat resistance mechanisms that type II FLT3 inhibitors suffer from (like Quizartinib). Also, inhibition of off-target tyrosine kinases that circumvent FLT3 inhibitors should be detected, such as FLT1, FLT4, PDGFRα, and PDGFRβ (like compound **27**), to elucidate the efficacy of these novel FLT3 inhibitors versus resistant AML cells. Besides, antiproliferative activity, cell cycle arrest, and apoptosis induction properties were examined specifically for compound **27**. In vivo studies should be carried out for compounds **26** and **27** to investigate their potent cytotoxicity versus FLT3 AML cells, their higher tolerability with no significant toxicity signs, their overall response rate, progression-free survival, and overall survival rate. Therefore, all those benzimidazole-based scaffolds had to be fully examined for their physicochemical properties, tolerability, cell cycle arrest, and apoptosis induction capabilities to facilitate the development of more potent and selective FLT3 inhibitors against various FLT3 AML cells. Moreover, the affinity of those benzimidazole-based FLT3 inhibitors to cause severe myelosuppression as an urgent side effect resulting from the inhibition of c-kit kinase; should be tested to depict the efficacy of those FLT3 inhibitors to target FLT3 AML cells with fewer and less urgent side effects.

## Conclusion & future perspective

Despite developing various FLT3 inhibitors, AML is classified as one of the most resistant and problematic cancer types to be treated with lower than 30% of investigated AML cases with a survival rate of 5 years or more. Numerous small structured FLT3 inhibitors have been approved for AML therapy. Starting from the first successful FDA-approved indole-based FLT3 inhibitor in 2017, Midostaurin opened the door for a new era for treating mutated FLT3 AML patients. Then, Gilteritinib was authorized in the next year to treat relapsed or refractory AML with mutated FLT3 types. However, Quizartinib was only approved for the FLT3-ITD-relapsed, or refractory AML investigated cases in Japan. Extra small structured FLT3 inhibitors are being investigated in clinical and preclinical studies. Resistance is a co-existing issue and is a fundamental obstacle in the fruitful development and clinical validation of these novel FLT3 inhibitors. Hence, FLT3 inhibitors targeting different mutated FLT3 types have been developed to alleviate specifically on-target mutation mechanisms. Yet, resistance mechanisms at the F691L gatekeeper residue persist in being a difficult type of mutation to target. Unfortunately, resistance mechanisms attenuate drug empathy to FLT3, resulting in poor clinical responses and a quick relapse. The new FLT3 inhibitor FF-10101 which covalently interacted with the C695 cysteine residue, is usually used to lessen on-target mutations. Notably, it was found that FF-10101 preserved its FLT3 inhibitory activity even in the presence of the F691L mutation, illustrating that irreversible FLT3 inhibitors could be effective in drug-resistant resistance mechanisms. A closer look at the mutation mechanisms of FLT3 inhibitors could give us a clear outline that greatly helps design combinatorial therapeutic clinical trials targeting identified mutation mechanisms. Additionally, the design of multi-targeted FLT3 inhibitors that circumvent complementary signaling cascades could also potentiate the therapeutic efficacy of these agents. AML heterogeneity is a constant and critical problem that affords extra mutations when a new therapeutic agent is evaluated. The most promising trend to counteract these new mutations is to finalize clinical studies with new candidates or combination therapeutic protocols and consequently be able to detect the possible mutation mechanisms that happened at relapse. If these mechanisms are known, and drug targets are identified, new therapeutic candidates can be developed to oppose these mutation mechanisms. However, the tractable mutation mechanisms targeted with available drugs and new combinatorial therapeutic protocols can be investigated in clinical trials. Identifying and developing novel FLT3 inhibitors targeting these new mutation mechanisms are fundamental to reducing the duration of the mitigation. These procedures are repeatable and gradually incremental but of high necessity for affording more powerful and unique strategies that are efficient enough to struggle against AML. Triggered by the previous research studies about FLT3 inhibition, we reviewed several classes of indole and other indole-based selective FLT3 inhibitors, including azaindole, oxindole, indazole, and benzimidazole based scaffolds highlighting their specific antiproliferative potential versus AML cells.

Notably, a methanone indole derivative, compound **4** displayed high potency, specificity, and selectivity as an FLT3 inhibitor towards FLT3-ITD-F691L with binding affinity (Kd) value equal to 114 ± 14 nM compared to Quizartinib, which has a Kd value of 340 ± 60 nM. Moreover, compound **6,** an azaindole-based scaffold, exhibited a tris kinase inhibitory agent versus FLT-3, GSK-3β, and VEGFR-2 with IC_50_ values of 18 nM, 9 nM, and 48 nM, respectively, and showed higher potency and selectivity versus Molm-14 AML cells with a cell viability percentage about 2.40% with 50 μM as compound **6** dosage. Furthermore, oxindole-based scaffold, compound **14** showed greater specify towards mutant FLT3 kinases subtypes, including FLT3(ITD- D835H), FLT3(ITD- F691L), and FLT3(D835H) with binding affinity values of 0.83 nM, 1.5 nM, and 1.3 nM, respectively, besides it exhibited promising antiproliferative activity against FLT3-ITD-MOLM-14 cells with IC_50_ value of 7.8 nM. Meanwhile, compound **15**, a 3-substituted oxime indirubin-based scaffold, displayed higher potency and circumvention activity against wt-FLT3, FLT3-ITD, and FLT3/D835Y with IC_50_ values of 0.87, 0.25, and 0.32 nM, respectively, and showed a higher antineoplastic activity versus wild FLT3 expressing MV4-11, MOLM15-wt, MOLM15-ITD, and MOLM15-ITD-D835Y mutant cells with GI_50_ values of 1.0, 4.88, 1.85, and 1.87 nM indicating more sensitivity to MOLM15-ITD cells than native kind of MOLM15 with the same manner of Gilteritinib. Compound **23**, the amino indazole derivative, strongly bonded with multiple kinases, especially FLT3, D835H-FLT3, ITD-FLT3, and KIT, with lower percent control proportions (% ctrl) of 0.15, 0.05, 0.1, and 0.05%, respectively, indicating its strong inhibitory capability. Also, compound **23** exhibited higher antiproliferative activity against FLT3-MOLM18, PDGFRa-T674M-Ba/F3, and Kit-T670I-Ba/F3 cells with EC_50_s equal to 5 nM, 16 nM, and 198 nM, respectively. Eventually, compound **27**, a benzimidazole-based scaffold, showed a selective inhibitory activity versus wild FLT3, FLT3-ITD, and FLT-TKD-D835Y mutated protein kinases with IC_50_s equal to 43.8 nM, 97.2 nM, and 92.5 nM, respectively. Compound **27** affords powerful antineoplastic activity versus FLT3-ITD acute myeloid leukemia cells, including MV4-16 and MOLM-18, with IC_50_s equal to 38.8 nM and 54.9 nM, respectively. Herein, within this review study, we highlight various indole and other indole-based FLT3 inhibitors even having only specific circumvention activity versus FLT3 or with other multiple kinase targets to afford a clear biological and chemical map about the indole-based kinase hampering agents that can be considered as efficient scaffolds utilized for proper FLT3-AML therapy, waiting for more evaluation through further physicochemical studies and clinical trials.

## Data Availability

Data and materials of our Figures or Tables are available with us because this is a review.
